# Dynamic of Composition and Diversity of Gut Microbiota in *Triatoma rubrofasciata* in Different Developmental Stages and Environmental Conditions

**DOI:** 10.3389/fcimb.2020.587708

**Published:** 2020-11-02

**Authors:** Yue Hu, Hanguo Xie, Minzhao Gao, Ping Huang, Hongli Zhou, Yubin Ma, Minyu Zhou, Jinying Liang, Jun Yang, Zhiyue Lv

**Affiliations:** ^1^ Joint Program of Pathobiology, Fifth Affiliated Hospital, Zhongshan School of Medicine, Sun Yat-sen University, Guangzhou, China; ^2^ Key Laboratory of Tropical Translational Medicine of Ministry of Education, Hainan Medical University, Haikou, China; ^3^ Key Laboratory of Tropical Disease Control (Sun Yat-sen University), Ministry of Education, Guangzhou, China; ^4^ Provincial Key Laboratory of Zoonosis Research, Fujian Center for Disease Control and Prevention, Fuzhou, China

**Keywords:** *Triatoma rubrofasciata*, gut microbiota, developmental stages, environmental conditions, *16S* rRNA gene sequencing

## Abstract

*Triatoma rubrofasciata* (*T. rubrofasciata*), one kind of triatomine insects, is the vector of *Trypanosoma cruzi* (*T. cruzi*), which lead to American trypanosomiasis. Although the gut microbiome may play an essential role in the development and susceptibility of triatomine, there is limited research on the gut microbiota of *T. rubrofasciata*. To elucidate the effect of the vector’s developmental stages and environmental conditions on the gut microbiome, we employed *16S* rRNA gene sequencing to profile the gut bacterial community diversity and composition of *T. rubrofasciata*. Significant shifts were observed in the overall gut microbe diversity and composition across the development of *T. rubrofasciata* and specific bacteria were detected in different stages. *Serratia* and *Burkholderia-Caballeronia-Paraburkholderia* were dominant in the 1^st^ nymphal stage, while the abundance of *Staphylococcus* was low in the 1^st^ nymphal stage. *Oceanicaulis* were undetectable in the adult stage and *Odoribacter* peaked in the 2^nd^ nymphal stage. Moreover, *Staphylococcus* was correlated negatively with *Serratia*. Likewise, the total gut microbiota diversity and composition of *T. rubrofasciata* differentiated significantly by environmental conditions. The ingestion of a bloodmeal increased alpha diversity of gut bacterial communities, and *Staphylococcus* was more abundant in laboratory-reared bugs whereas *Enterococcus* enriched in wild-caught bugs. Furthermore, *Pantoea* was negatively correlated with *Staphylococcus*, and positively related to *Bacillus* only. The phylogenetic Investigation of Communities by Reconstruction of Unobserved States (PICRUSt) algorithm showed obvious metagenomic functional differences by environmental conditions, and Chagas disease relevant pathway was enriched in wild-caught *T. rubrofasciata*.

## Introduction

American trypanosomiasis, also named Chagas disease, is a vector-borne disease for which the causative agent is the protozoan parasite *Trypanosoma cruzi* (*T. cruzi*), which is mainly transmitted by triatomines. Chagas disease is also one of the chronic, systemic and neglected tropical diseases (NTDs); with 8 million people infected worldwide, mostly in Latin America, at least 70 million people are at risk of contagion (Gourbière et al., 2012; Orantes et al., 2018; WHO, 2020). Chagas disease had been commonly recorded in Latin American and Caribbean (LAC) region in the past decades, however, it is being increasingly reported as an emerging infectious disease in North America, Europe and the Indo-Pacific region because of the frequent international migration of population and global invasion of the widespread kissing-bug (Hotez et al., 2012; Dujardin et al., 2015). Furthermore, Chagas disease ranks near the top in terms of annual deaths and disability-adjusted life years (DALYs) lost among all NTDs in the Americas because of its highly debilitating chronic course with alteration of the cardiovascular, digestive and nervous systems (Hotez et al., 2012; Sassera et al., 2013). Since a vaccine or effective treatment for Chagas disease is still unavailable, adequate prevention and control of the disease may be achieved by control of the vectors.

Currently, triatomine insects are composed of 151 species that are grouped into 17 genera and organized into 5 tribes, that is, Aberproseniini, Bolboderini, Cavernicolini, Rhodiniini and Triatomini (Galvão and Justi, 2015; Vieira et al., 2018). Most triatomine species occur in the Americas, while six species belonging to the genus *Linshcosteus* are found in India; moreover, the species of the genus *Triatoma* are distributed in Africa, the Middle East, South-East Asia and the Western Pacific (Vieira et al., 2018).

*Triatoma* is one of the most diverse genera, including the species *Triatoma* *rubrofasciata* (*T. rubrofasciata*), which is widespread throughout southern China, such as in Guangdong, Guangxi, Hainan and Taiwan (Ibarra-Cerdeña et al., 2009; Huang et al., 2018). *T. rubrofasciata* is a domiciliated species with urban characteristics and exists in close association with rodents that act as reservoirs of *T. cruzi*. It is naturally infected by *T. cruzi* as well as *Trypanosoma conorrhini*, which is pathogenic to *Rattus rattus* but not to humans (Braga et al., 1998; Cortéz and Gonçalves, 1998). Due to the wide distribution of *T. rubrofasciata* in South China, which suggests the possibility of introduction and transmission of Chagas disease in China, further study of this vector is urgently required.

Triatomines are hemipteran (true) bugs and obligate hematophagous insects with five nymphal stages before the egg reaches adulthood (Gourbière et al., 2012; Oliveira et al., 2018; Vieira et al., 2018). Because both nymphs and adults feed on the blood of vertebrates, so they may become infected and are likely to transmit *T. cruzi* after they ingest blood from infected mammalian hosts. Once the parasites arrive at the gut of the triatomine, they multiply and are able to infect a new host during a subsequent blood meal; they also come in contact with the local gut microbiota and avoid detrimental interactions with the microbiome to survive and develop inside the bug gut (Azambuja et al., 2005; Gourbière et al., 2012; Oliveira et al., 2018).

Various studies of triatomine gut microbes have been conducted, and to date, more than 57 species of cultivable bacteria have been identified (Lima et al., 2018). Previous studies have shown that the most common bacteria in eight species of wild-caught and laboratory-reared triatomines were Gram-negative rods (Azambuja et al., 2005). Interestingly, bacteria of the genus *Rhodococcus* in the triatomine gut are believed to play an important role in the metabolism of the vector, such as by participating in the synthesis of group B vitamins or by being digested by the bugs directly to provide missing nutrients (Sassera et al., 2013). Moreover, the most attractive aspect is the host-symbiont relationship between triatomines and *Rhodococcus*; since *Rhodococcus* bacteria can be easily cultured and genetically modified to harm the pathogen in vector gut, they are probably suitable tools for the control of trypanosomiasis (Sassera et al., 2013). Another study found that the SM365 and RPH strains of *Serratia marcescens*, which is a common symbiont of various triatomine species, exhibit trypanolytic activity toward several *T. cruzi* strains (Azambuja et al., 2004).

However, previous studies of the triatomine gut microbiome have been based mostly on the isolation and identification of cultivable bacteria, did not reflect the relative abundances of these species under natural conditions, and inevitably missed many taxa that could not be cultivated (da Mota et al., 2012; Oliveira et al., 2018). On the other hand, cultivation-independent methods, such as high-throughput DNA sequencing, allow fast and accurate description of bacterial diversity, especially for uncultivable microbes, which are impossible to detect with cultivation-dependent methods (da Mota et al., 2012; Oliveira et al., 2018).

To date, *16S* ribosomal RNA (rRNA) gene sequencing has been applied in the characterization of the gut microbiota in some triatomine species (Gumiel et al., 2015; Díaz et al., 2016; Dumonteil et al., 2018; Oliveira et al., 2018; Orantes et al., 2018; Rodríguez-Ruano et al., 2018); to the best of our knowledge, the gut microbiome of *T. rubrofasciata* has not yet been investigated. Hence, in this study, we applied this technology to determine and compare the relative abundance of both cultivable and uncultivable bacteria in the gut of *T. rubrofasciata* from 1^st^ instar nymphs to adults for the first time and tried to elucidate the potential interactions between the vector and colonizing bacteria during vector development. In addition, we used *16S* rRNA gene sequencing to examine the gut flora of wild-caught and laboratory-reared *T. rubrofasciata* for the first time to explore and compare the diversity and composition of the gut microbes of triatomines and reveal the correlations between environmental conditions and specific gut microbiota profiles.

## Materials and Methods

### Bug Collection and DNA Extraction

The four wild adult triatomines used in this study were captured in Huping village (E 117°38′, N 24°39′), Fengshan town, Hua’an county, Zhangzhou city, Fujian province, China, by technicians from the Fujian Center for Disease Control and Prevention, China, in August 2017. A set of 45 randomly selected laboratory-reared triatomines at each developmental stage (1^st^ to 5^th^ instar nymphs plus male and female adults) were reared from eggs of local *T. rubrofasciata* caught in Huping village between July and August 2017.

DNA extraction from the entire gut of individual bugs was performed by using a QIAamp DNA Mini Kit (cat. no. 51306, QIAGEN, Hilden, Germany) according to the manufacturer’s protocols. The quality and quantity of the DNA were determined by agarose gel electrophoresis and a Nanodrop 2000 spectrophotometer (Thermo Scientific, Waltham, MA, USA), respectively. Then, the extracted DNA was partly diluted to a concentration of 1 ng/μL and stored at -20°C until use.

### Detection of *T. rubrofasciata* Feeding Sources and *T. cruzi* Infection

To determine the feeding sources of wild-caught and laboratory-reared adult triatomines, we used the primer set BM1/BM2 (**Table 1**) synthesized by Sangon Biotech (Shanghai, China) to amplify the mitochondrial cytochrome *b* (*cytb*) gene of all vertebrates. Polymerase chain reactions (PCRs) were performed in a total volume of 50 μL containing approximately 30 ng of DNA template, 1.1 × Golden Star T6 Super PCR Mix (Tsingke, China) and 0.4 μmol/L each primer. Sterile water was used as a negative control to avoid contamination during PCR. Amplifications were performed in a Bio-Rad PCR C1000 Touch instrument (Bio-Rad, USA), with an initial denaturation at 98°C for 2 min, followed by 35 cycles of denaturation at 98°C for 10 s, annealing at 56°C for 30 s, and extension at 72°C for 15 s and a final extension at 72°C for 3 min.

**Table 1:** Primers used to amplify the specific genes in this study.

Gene target	Primer name	Forward primer (5′→3′)	Reverse primer (5′→3′)	Expected length (bp)
Vertebrate mtDNA *cytb*	BM1/BM2	CCCCTCAGAATGATATTTGTCCTCA (Boakye et al., 1999)	CCATCCAACATCTCAGCATGATGAAA (Boakye et al., 1999)	358
*Trypanosoma cruzi* nDNA satellite repeat	TCZ1/TCZ2	CGAGCTCTTGCCCACACGGGTGCT (Moser et al., 1989)	CCTCCAAGCAGCGGATAGTTCAGG (Moser et al., 1989)	188
*Trypanosoma cruzi* minicircle kDNA	Tc121/Tc122	AAATAATGTACGGG(T/G)GAGATGCATGA (Wincker et al., 1994)	GGTTCGATTGGGGTTGGTGTAATATA (Wincker et al., 1994)	330

To determine the natural infection status of wild-caught triatomines, the *T. cruzi*-specific TCZ1/TCZ2 nuclear satellite repeat and Tc121/Tc122 minicircle fragment were amplified with the primers listed in **Table 1**. The mixture and conditions used to amplify these two targets were the same as those for *cytb* except that the annealing temperature of the nuclear satellite repeat was different and was set as 60°C.

All PCR products were observed by electrophoresis on 2% agarose gels stained with ethidium bromide, and then the products of all positive reactions were purified using an Agarose Gel DNA Extraction Kit (Takara, Japan). Sequencing reactions were performed using the primers listed in **Table 1** in an Automated DNA Analyzer (ABI 3730XL, Applied Biosystems, Foster City, CA, USA) with the BigDye Terminator v3.1 Cycle Sequencing Kit (cat. no. 4337457, Applied Biosystems, Foster City, CA, USA) by Tsingke Biotechnology Ltd., Co. (Guangzhou, China). Then, the sequences were submitted to the GenBank database for homology searches using BLAST^1^.

### *16S* rRNA Gene Amplification, Library Construction, and Next-Generation Sequencing

The V3-V4 variable regions of the *16S* rRNA gene were amplified with the primers 343F (5’-TACGGRAGGCAGCAG-3’) and 798R (5’-AGGGTATCTAATCCT-3’) in combination with the barcoded primers and Takara Ex Taq DNA polymerase (cat. no. RR001Q, Takara, Japan), while the diluted DNA was used as a template. The quality of the PCR amplicon products and the relative intensity of the bands were determined by agarose gel electrophoresis, and then, Agencourt AMPure XP beads (cat. no. A63881, Beckman Coulter, Brea, CA, USA) were applied to purify the amplicon, and another round of PCR was subsequently performed. After purification with Agencourt AMPure XP beads once again, the final amplicon was quantified by a Qubit^®^ dsDNA HS Assay Kit (cat. no. Q32852, Life Technologies, Waltham, MA, USA). Purified amplicons from each sample were pooled together in equal amounts for next-generation sequencing on the Illumina MiSeq platform following the manufacturer’s guidelines.

### Quality Control of Sequencing Data and Bacterial Classification

Raw sequencing data were obtained in FASTQ format. Paired-end reads were then preprocessed with Trimmomatic software^2^ (Bolger et al., 2014) to detect and cut off ambiguous bases from the N terminus. Low quality sequences with average quality scores lower than 20 were also removed using the sliding window trimming approach. Then, paired-end reads were assembled by Fast Ligation-Based Automatable Solid-Phase High-Throughput (FLASH) software (version 1.2.11) (Reyon et al., 2012). The parameters for assembly were set as 10 base pairs (bp) of minimum overlap, 200 bp of maximum overlap and 20% maximum mismatch rate. Furthermore, reads with ambiguous, homologous sequences and a total length of less than 200 bp were abandoned, while reads with 75% bases above Q20 were retained. Next, reads with chimeras were detected and removed by Quantitative Insights Into Microbial Ecology (QIIME) software (version 1.8.0) (Caporaso et al., 2010).

Clean reads were subjected to primer sequence removal and clustering to generate operational taxonomic units (OTUs) (Blaxter et al., 2005) using Vsearch software^3^ (Rognes et al., 2016) with a 97% sequence similarity cutoff (equal to the bacterial species level). The representative read was chosen from each OTU by selecting the most abundant sequence using the QIIME package. All high-quality representative sequences were annotated and blasted against the Silva database (version 123) on the basis of the Ribosomal Database Project (RDP) classifier with the confidence threshold set as 70% (Wang et al., 2007).

Sequences identified as DNA from non-bacterial sources, such as chloroplasts, mitochondria, Archaea, and Eukarya, as well as singletons, were filtered out. Then, the rarefied OTU table was applied to illustrate the composition of the gut bacteria in each bug and for further analysis.

### Alpha Diversity and Beta Diversity

To estimate within-sample diversity, alpha diversity estimators, such as the number of observed species, Chao1 richness estimator, Shannon-Wiener index, Good’s coverage estimator and phylogenetic diversity index, were obtained by using Mothur^4^ (Schloss et al., 2009) for each individual. The number of observed species indicated the number of OTUs that were actually observed, while the Chao1 richness estimator was used to estimate the number of OTUs that were actually present in the bacterial community. The Shannon-Wiener index reflected both the richness and the evenness of species in the community; the higher the value was, the higher the diversity of the community. Sample coverage was revealed by the Good’s coverage estimator, and the depth of sequencing substantially covered all the species in the sample if the value was close to one. The phylogenetic diversity index based on random sampling of OTUs indicated the evolutionary distance relationship among OTUs; the higher the value was, the greater the evolutionary distance of the species.

Beta diversity, that is, between-sample diversity, was then monitored with principal coordinate analysis (PCoA) and nonmetric multidimensional scaling (NMDS), two kinds of ordination analyses, based on weighted UniFrac distance metrics or Bray-Curtis distance metrics. Moreover, one-way analysis of similarity (ANOSIM) was performed to assess whether the differences among the groups identified by NMDS were significant with 1,000 Monte Carlo permutation tests. In addition, the relative levels of gut bacterial community dispersal from each group were visualized by bar plots at both the phylum level and the genus level.

### Statistical Analysis

One-way analysis of variance (ANOVA) or Student’s *t* test was performed to distinguish differences in gut microbial communities among multiple groups or between two groups, respectively, and differences were considered statistically significant at a *p*-value lower than 0.05. Linear discriminant analysis (LDA) coupled with effect size (LEfSe) measurement^5^ (Segata et al., 2011) was applied to identify gut microbiome that were distinct from different groups. To identify the key signature gut flora of triatomines, a random forest (RF) classification model was built to estimate the importance of the top 30 dominant genera with the mean decrease gini, a measure applied to rank each genus in the model. Finally, Spearman correlation coefficients among the top 30 predominant bacterial genera were calculated to demonstrate the relationships among these genera. Unless otherwise stated, statistical analyses and plotting were carried out using R software (version 3.5.1).

### Functional Profile Prediction for Gut Bacterial Communities

The functional profiles of gut bacterial communities were predicted with Phylogenetic Investigation of Communities by Reconstruction of Unobserved States (PICRUSt)^6^ (Langille et al., 2013) on the basis of *16S* sequencing data annotated by the Greengenes reference database^7^ (DeSantis et al., 2006). Then, the predicted functions in combination with functional categorization were obtained by mapping the normalized OTU data in the Kyoto Encyclopedia of Genes and Genomes (KEGG) database^8^. The differences between samples and groups were calculated by the Kruskal-Wallis (KW) rank sum test and visualized by a heatmap in level 3 of KEGG pathways.

## Results

### Identification of Feeding Sources and Natural Infection

Using the primer set BM1/BM2, we successfully amplified the *cytb* gene of vertebrates from the digestive tract of laboratory-reared adult triatomines, obtaining the characteristic 358-bp product, but this amplification failed in wild-caught triatomines. The feeding source of the laboratory-reared triatomines was identified as *Mus musculus*, as the triatomines were fed on the blood of ICR mice in our laboratory. Additionally, neither the *T. cruzi*-specific TCZ1/2 nuclear satellite repeat nor the Tc121/122 minicircle fragment was amplified from the gut of wild-caught triatomines, which suggested that the four bugs did not host *T. cruzi*.

### Summary of High-Throughput Sequencing Data

A total of 1,479,154 high-quality sequences were yielded using *16S* rRNA gene amplicon sequencing from 49 extracted DNA samples of the triatomine gut, with an average of 30,186 reads per sample after quality and abundance filtering, and the average sequence length was 438 bp. Among these reads, 1,765 OTUs were identified at a sequence similarity threshold of 97% (**Supplementary Table 1**). The high value of Good’s coverage index in all the triatomine samples indicated that the sequencing depth was sufficient for profiling of the bacterial communities present (**Supplementary Table 2**).

### Dynamic Alteration in the Gut Microbiota Across *T. rubrofasciata* Developmental Stages

**Figure 1:**
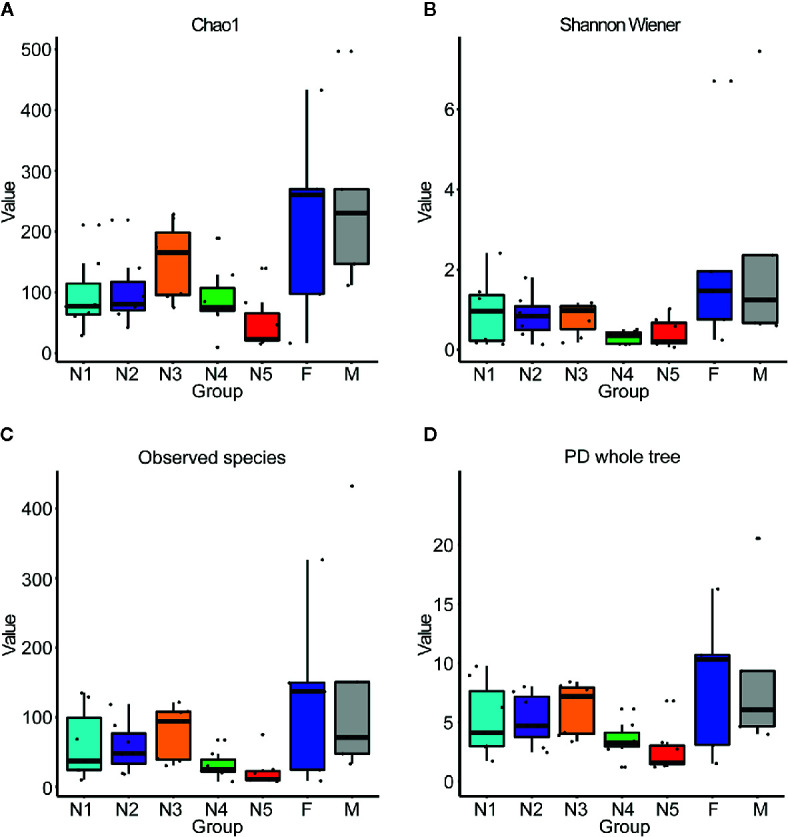
Box plots of *16S* rRNA gene sequences obtained from gut samples of *T. rubrofasciata* across developmental stages based on alpha diversity analyses. Each column represents one group (N1, 1^st^ stage nymphs; N2, 2^nd^ stage nymphs; N3, 3^rd^ stage nymphs; N4, 4^th^ stage nymphs; N5, 5^th^ stage nymphs; F, female adult; M, male adult). The top and bottom whiskers indicate the maximum and minimum values, respectively, and the hyphen represents the median value. **(A)** Chao1. **(B)** Shannon Wiener. **(C)** Observed species. **(D)** PD whole tree.

**Figure 2:**
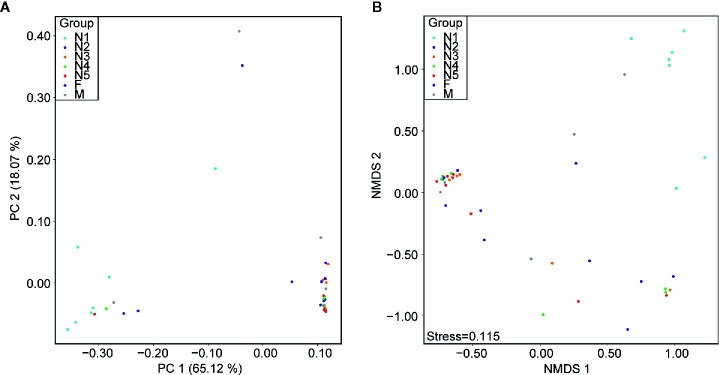
Beta diversity differences in *T. rubrofasciata* gut samples across developmental stages on the basis of *16S* rRNA gene sequencing (N1, 1^st^ stage nymphs; N2, 2^nd^ stage nymphs; N3, 3^rd^ stage nymphs; N4, 4^th^ stage nymphs; N5, 5^th^ stage nymphs; F, female adult; M, male adult). Each point represents an individual. **(A)** Principal coordinate analysis (PCoA) plot of weighted UniFrac distances. **(B)** Nonmetric multidimensional scaling (NMDS) plot of Bray-Curtis distance.

First, alpha diversity estimators were used to reveal marked differences in the richness and diversity of species in the community during triatomine development. Statistically significant differences were observed in the Chao1 index, Shannon-Wiener index, observed species and phylogenetic diversity index among the bug development stages. All four of the alpha diversity estimates increased in young nymphs (1^st^-3^rd^ stages) but decreased obviously from 4^th^ stage nymphs, reaching the lowest value in nymphs of the fifth instar; however, the value rebounded to the highest value in adults, especially in the female adults (**Figure 1**, KW test, *p* < 0.05 for all comparisons), which indicated that the gut bacteria of *T. rubrofasciata* were affected by aging, and the gut communities in adults were more rich and even than those at earlier stages. Then, PCoA plots based on weighted UniFrac distances demonstrated that the composition of the bug gut flora was related to the development stage, with 65.12% and 18.07% variation explained by principal component (PC) 1 and PC 2, respectively; 1^st^ stage nymphs were distant from the other nymphs and adults, and this result was mainly driven by the largest differences in the abundances of *Staphylococcus* and *Serratia* between 1^st^ stage nymphs and the others (**Figure 2A**). Obvious separation of nymphs of the first instar was also found in the NMDS plot on the basis of Bray-Curtis dissimilarities; moreover, the separation of 1^st^-2^nd^ stage nymphs from older nymph stages and adults became evident, although older nymph stages and adults seemed closely related to each other, samples were still clustered according to developmental stage (**Figure 2B**). The stress value of NMDS in combination with the one-way ANOSIM results (R = 0.1822, *p* = 0.001) suggested that the grouping of NMDS was reliable. Overall, diversity analyses indicated that the gut microbes of *T. rubrofasciata* differ with age, in both the richness and evenness of the community in the microbiota structure.

**Figure 3:**
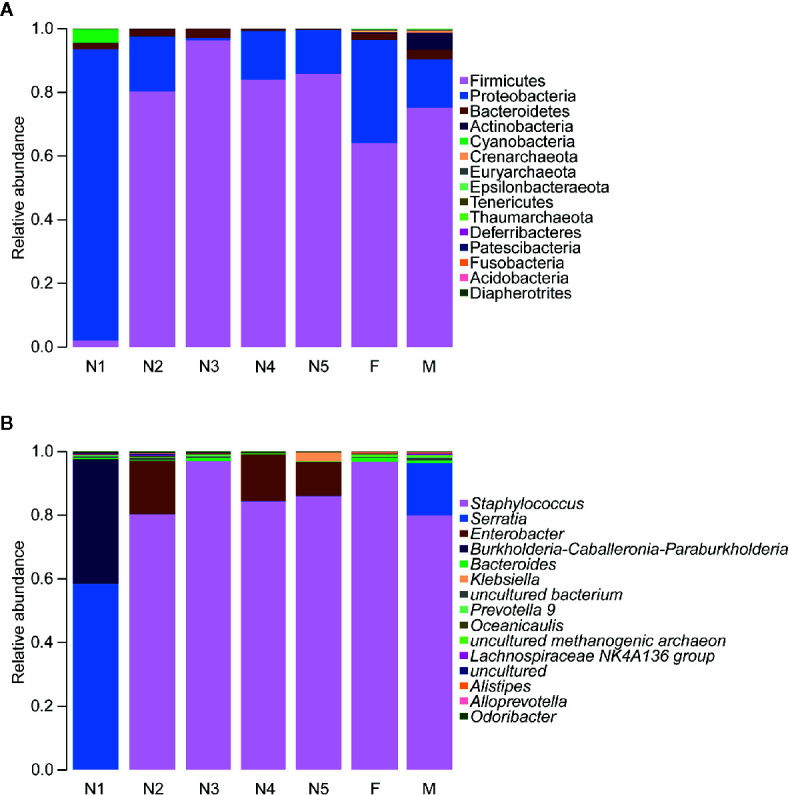
Relative abundances of the 15 most important gut microbiota constituents at the phylum level **(A)** and genus level **(B)** across development stages of *T. rubrofasciata*, as assessed by *16S* rRNA sequencing. Each column represents the composition of the microbial taxa in one group (N1, 1^st^ stage nymphs; N2, 2^nd^ stage nymphs; N3, 3^rd^ stage nymphs; N4, 4^th^ stage nymphs; N5, 5^th^ stage nymphs; F, female adult; M, male adult).

**Figure 4:**
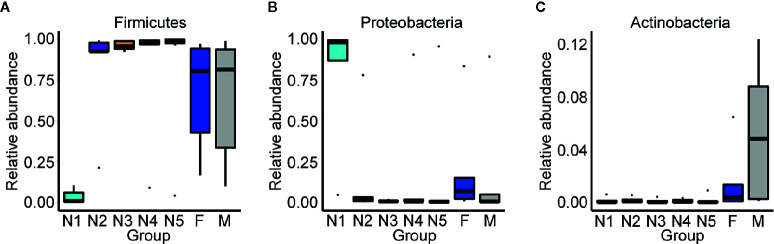
Relative abundances of the significantly altered 15 most important gut phyla across developmental stages of *T. rubrofasciata*. Each column represents one group (N1, 1^st^ stage nymphs; N2, 2^nd^ stage nymphs; N3, 3^rd^ stage nymphs; N4, 4^th^ stage nymphs; N5, 5^th^ stage nymphs; F, female adult; M, male adult). The top and bottom whiskers indicate the maximum and minimum values, respectively, and the hyphen represents the median value. **(A)** Firmicutes. **(B) **Proteobacteria. **(C)** Actinobacteria.

**Figure 5:**
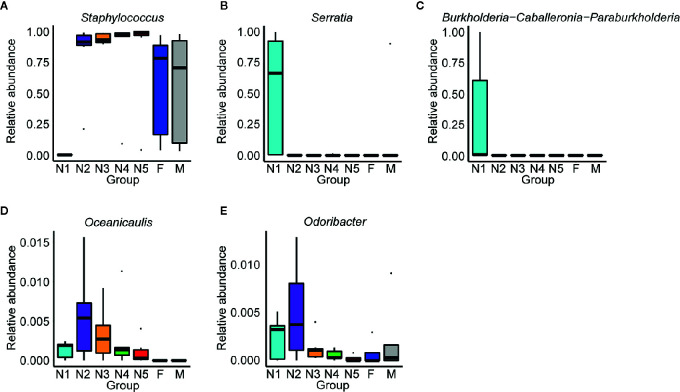
Relative abundances of the significantly altered 15 most important gut genera across developmental stages of *T. rubrofasciata*. Each column represents one group (N1, 1^st^ stage nymphs; N2, 2^nd^ stage nymphs; N3, 3^rd^ stage nymphs; N4, 4^th^ stage nymphs; N5, 5^th^ stage nymphs; F, female adult; M, male adult). The top and bottom whiskers indicate the maximum and minimum values, respectively, and the hyphen represents the median value. **(A)** *Staphylococcus*. **(B)** *Serratia*. **(C)** *Burkholderia-Caballeronia-Paraburkholderia*. **(D)** *Oceanicaulis*. **(E)** *Odoribacter*.

Subsequently, bar plots were generated based on the relative abundances of the top 15 dominant gut bacteria at the phylum level (**Figure 3A**) and the genus level (**Figure 3B**) in different groups to observe the alteration in the gut microbiota composition during the development of *T. rubrofasciata*. Meanwhile, one-way ANOVA was performed to identify significantly altered gut bacterial phyla and genera across the developmental stages. Firmicutes, Proteobacteria, Bacteroidetes and Actinobacteria were the prevalent gut bacterial phyla of the bugs, accounting for more than 95% of the average relative abundances at different ages of *T. rubrofasciata*. Among these phyla, the average abundances of Firmicutes, Proteobacteria and Actinobacteria were altered significantly (*p* < 0.05, **Supplementary Table 3**). The average abundance of Proteobacteria was the highest in the 1^st^ nymphal stage, while the average abundance of Firmicutes was the lowest. However, the opposite result was obtained in the 3^rd^ nymphal stage (**Figures 4A, B**). Furthermore, Actinobacteria, which was almost undetectable in nymphs, increased markedly in adults, particularly in male adults (**Figure 4C**). Moreover, *Staphylococcus*, *Serratia*, *Enterobacter*, *Burkholderia-Caballeronia-Paraburkholderia* and *Bacteroides* were the 5 most important gut bacterial genera in triatomines (**Figure 3B**); nevertheless, the average relative abundance of *Staphylococcus* was low in nymphs of the first instar, whereas *Serratia* was significantly more abundant in 1^st^ stage nymphs (**Figures 5A, B**, *p* < 0.05; see also **Supplementary Table 4**). Interestingly, *Burkholderia-Caballeronia-Paraburkholderia*, which showed the highest average abundance in the 1^st^ nymphal stage, as well as *Oceanicaulis*, was undetectable in the adult stage (**Figures 5C, D**, *p* < 0.05; see also **Supplementary Table 4**). Additionally, the average abundance of *Odoribacter* peaked in the 2^nd^ nymphal stage (**Figure 5E**, *p* < 0.05; see also **Supplementary Table 4**). Taken together, the results showed that not only gut bacterial genera but also gut bacterial phyla changed significantly across *T. rubrofasciata* developmental stages.

**Figure 6:**
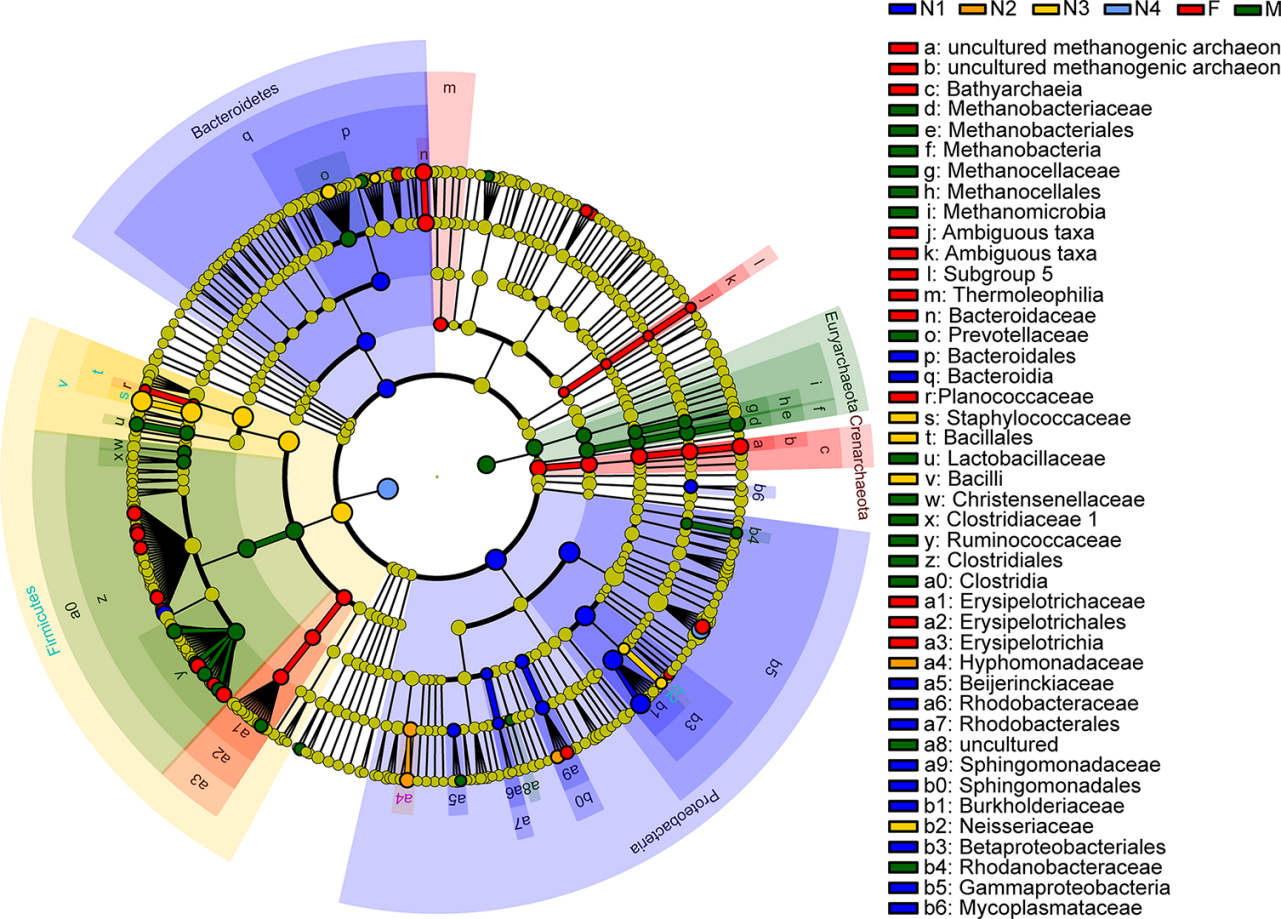
Cladogram showing discriminated taxa at different ages of *T. rubrofasciata* (N1, 1^st^ stage nymphs; N2, 2^nd^ stage nymphs; N3, 3^rd^ stage nymphs; N4, 4^th^ stage nymphs; N5, 5^th^ stage nymphs; F, female adult; M, male adult). Regions with different colors represent different groups. Different colored nodes in the branches represent the microbial groups that play an important role in the corresponding groups, whereas yellow nodes indicate bacterial groups that are nonsignificant in all groups.

Next, LEfSe was applied to identify taxa from the phylum to genus level that were distinct among the developmental stages of triatomines. The cladogram in **Figure 6** shows that *Burkholderia-Caballeronia-Paraburkholderia*, a bacterium from the class Gammaproteobacteria, phylum Proteobacteria, was markedly enriched in the 1^st^ nymphal stage, while *Oceanicaulis* and *Altererythrobacter*, bacteria belonging to the class Alphaproteobacteria, phylum Proteobacteria, were significantly associated with the 2^nd^ nymphal stage. *Staphylococcus*, within the phylum Firmicutes, was identified as the key marker of the 3^rd^ nymphal stage; because it showed higher abundance in nymphs of the third instar than in other stages. Similarly, *Enterobacter*, a member of the class Gammaproteobacteria, phylum Proteobacteria, was linked to the 4^th^ nymphal stage due to its highest average abundance in that stage. *Bacteroides*, in addition to some members from the order Clostridiales, phylum Firmicutes, was the significant bacterial genus related to the adult stage. Nevertheless, no discriminative gut microbiome was found in the 5^th^ nymphal stage.

**Figure 7:**
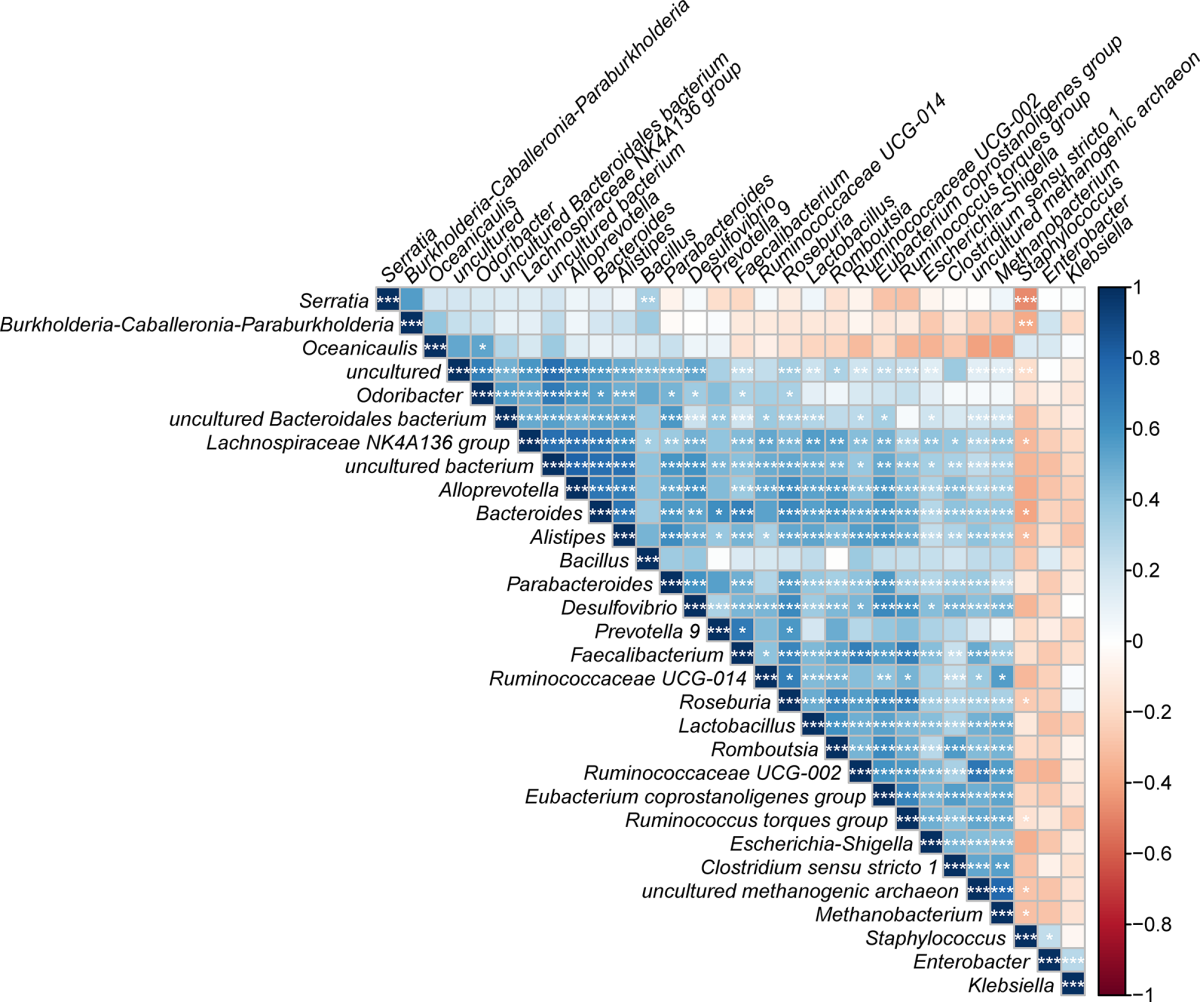
Correlation plot showing the relationship among the top 30 predominant gut bacteria across developmental stages of *T. rubrofasciata*. The correlations between the bacteria are indicated by colors; blue indicates positive correlation, while red indicates negative correlation, with a darker color indicating a stronger correlation (*p < 0.05, **p < 0.01, ***p < 0.001).

Finally, the correlation analysis results for the top 30 dominant bacterial genera were plotted (**Figure 7**). Notably, a majority of the bacteria were significantly positively related to each other, which revealed a symbiotic relationship of the gut microbiota in *T. rubrofasciata*. Even so, most negative correlations were found between the 5 most abundant gut bacteria and the remaining bacteria; most importantly, *Staphylococcus* was negatively correlated with *Serratia* (*p* < 0.001).

### Comparison of the Gut Microbiota in Wild-Caught and Laboratory-Reared *T. rubrofasciata*

**Figure 8:**
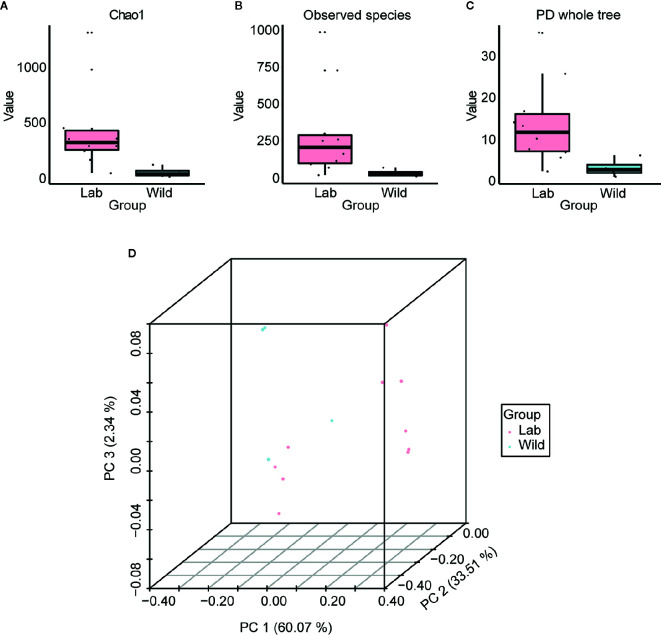
Alpha diversity and beta diversity of laboratory-reared and wild-caught *T. rubrofasciata*. **(A)** Chao1. **(B)** Observed species. **(C)** Phylogenetic diversity index. **(D)** Principal coordinate analysis (PCoA) plot of weighted UniFrac distances.

Based on the boxplot of Chao1, observed species and phylogenetic diversity, we found that the alpha diversity of wild-caught *T. rubrofasciata* was significantly lower than that of laboratory-reared *T. rubrofasciata* (**Figures 8A–C**, KW test, *p* < 0.05 for all comparisons); because the wild-caught triatomines had not ingested a blood meal before we captured them. In addition, differences in gut bacterial communities between wild-caught and laboratory-reared insects were visualized by a three-dimensional PCoA plot on the basis of weighted UniFrac distances. A clear separation was observed between the gut microbiome of wild-caught and laboratory-reared insects, with all three variables together explaining 95.92% of the total variance (**Figure 8D**).

**Figure 9:**
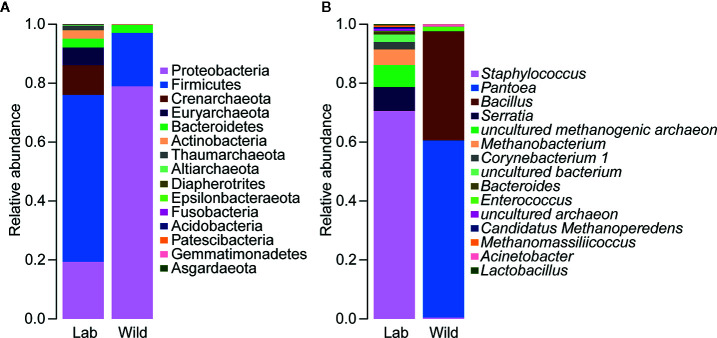
Relative abundances of the 15 most important gut microbiota constituents of laboratory-reared and wild-caught *T. rubrofasciata* at the phylum level **(A)** and genus level **(B)**, as assessed by *16S* rRNA sequencing.

Alterations in the gut flora were determined based on the relative proportions of different taxa, with a sharp reduction in the relative abundance of Proteobacteria (*p* < 0.05) and an increase in the abundance of Firmicutes, but the difference between laboratory-reared bugs and wild-caught bugs at the phylum level was not significant (**Figure 9A**; see also **Supplementary Table 5**). At the genus level, significant differences in bacterial abundances of the 15 most important genera were observed only in *Staphylococcus* and *Enterococcus*. The former was more abundant in laboratory-reared bugs than in wild-caught bugs, while the latter was relatively highly enriched in wild-caught bugs (**Figure 9B**, *p* < 0.05; see also **Supplementary Table 6**).

**Figure 10:**
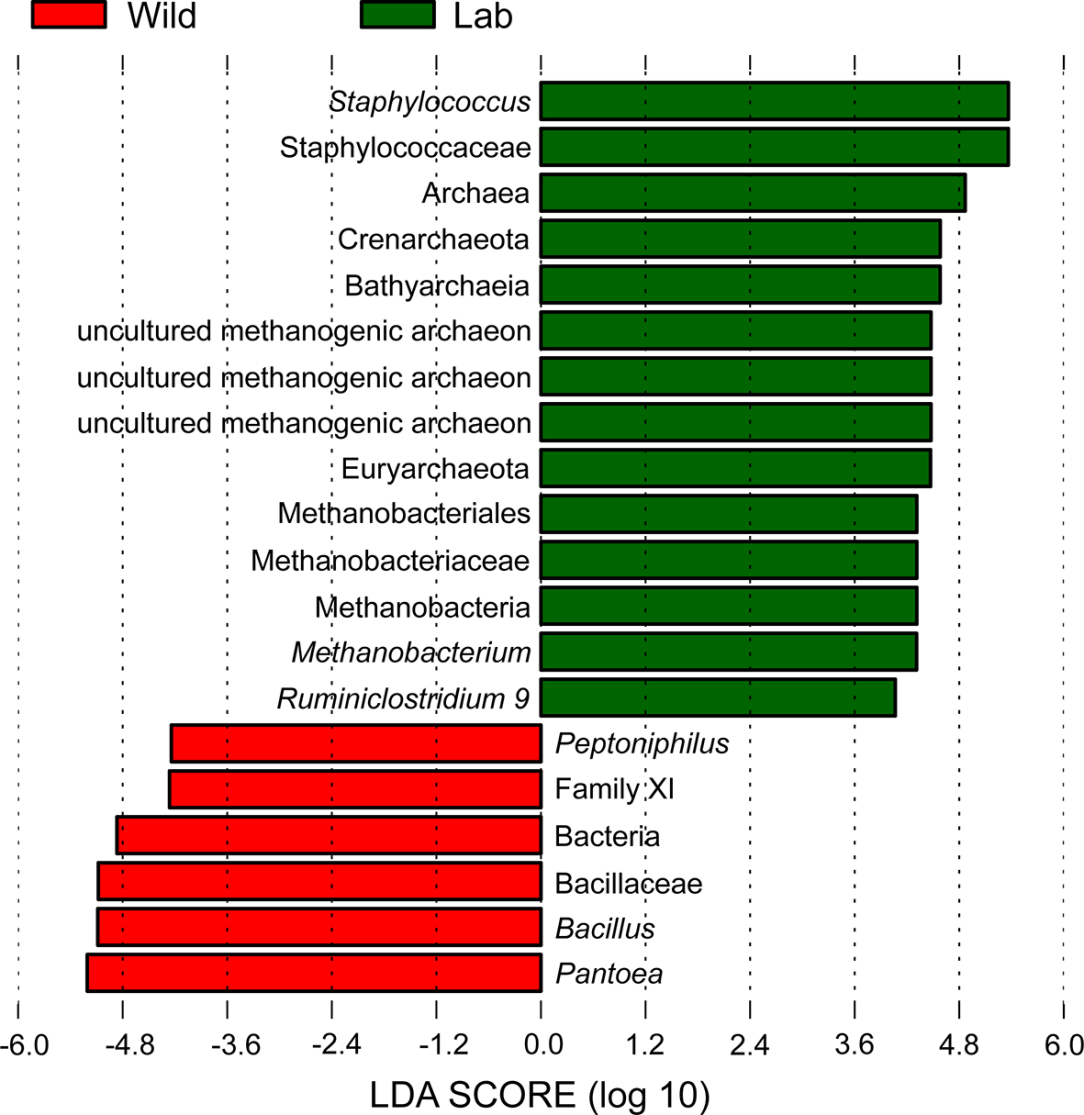
Histogram with LDA scores (threshold > 4) showing differentially abundant gut bacteria between laboratory-reared and wild-caught *T. rubrofasciata*. Taxa highlighted in green are overrepresented in laboratory-reared bugs, while those in red are overrepresented in wild-caught bugs.

**Figure 11:**
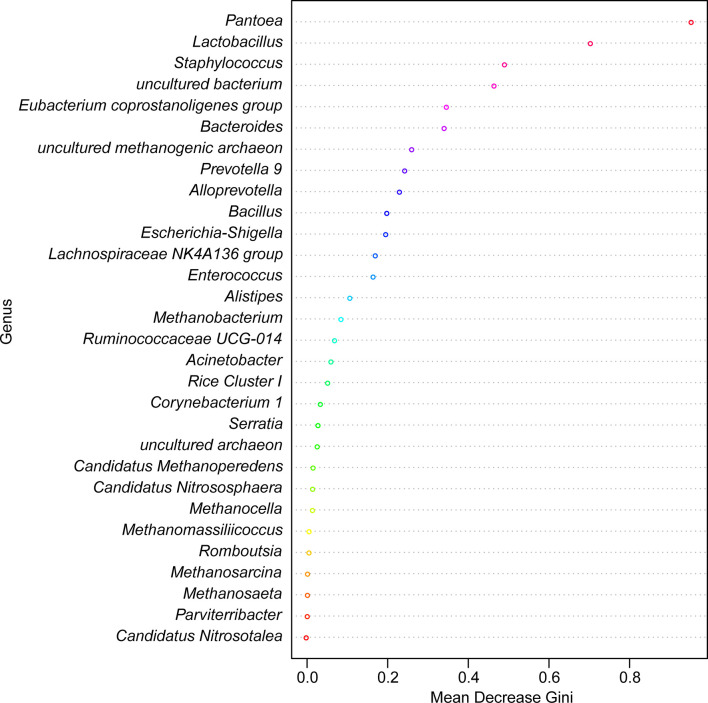
Random forest model classifying laboratory-reared and wild-caught *T. rubrofasciata* at the genus level. The importance levels are represented by the data for the mean decrease gini. Various bacterial genera are indicated by corresponding colors.

LEfSe identified 20 biomarkers, of which *Pantoea*, belonging to the phylum Proteobacteria, was markedly associated with wild-caught insects because of its absence in laboratory-reared insects, whereas *Staphylococcus*, within Firmicutes phylum, was closely linked with laboratory-reared insects (**Figure 10**). To establish a prediction model to distinguish laboratory-reared triatomines from wild-caught triatomines according to the abundance of genera as measured in the gut, we applied machine learning combined with RF analysis. The results demonstrated that the genera *Pantoea* and *Lactobacillus* were the variables with the highest importance in the model, which indicated that these two taxa had the strongest prediction power (**Figure 11**).

**Figure 12:**
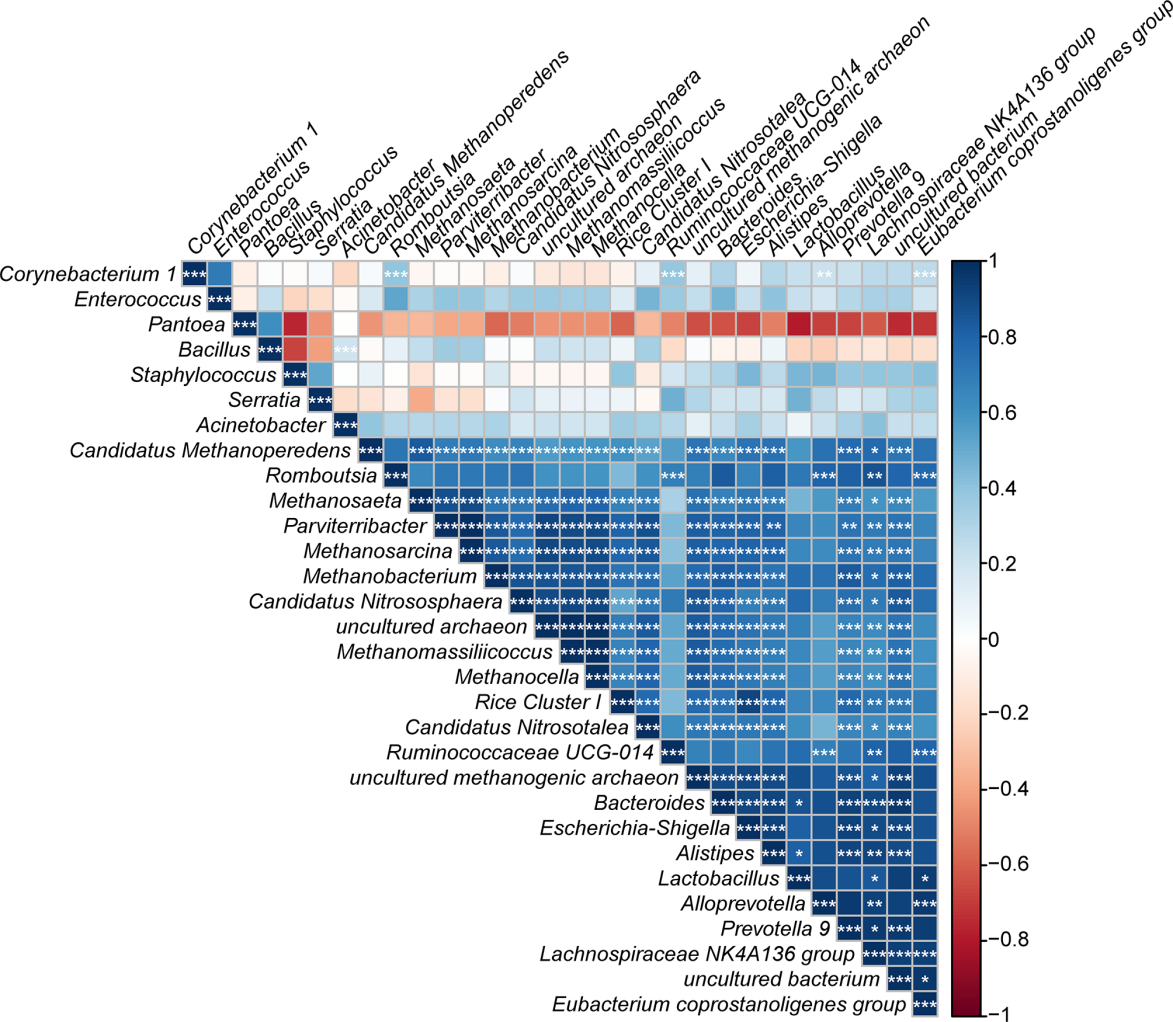
Correlation plot showing the relationship among the top 30 predominant gut bacteria in laboratory-reared and wild-caught *T. rubrofasciata*. The correlations between the bacteria are indicated by colors; blue indicates positive correlation, while red indicates negative correlation, and a darker color indicates a stronger correlation (*p < 0.05, **p < 0.01, ***p < 0.001).

Using the calculated Spearman correlation coefficients for the top 30 dominant gut microbes at the genus level, we observed both positive and negative correlations among these microbes, with the former being more common (**Figure 12**). Interestingly, *Pantoea* was negatively correlated with *Staphylococcus* and other taxa and positively related to *Bacillus* only (*p* > 0.05).

In addition, to predict the functional differences in the gut bacterial community between laboratory-reared and wild-caught *T. rubrofasciata*, a hierarchical clustering heat map was plotted by utilizing the PICRUSt algorithm to map the KEGG pathways (**Supplementary Figure 1**). The results demonstrated that pathways associated with metabolism of amino acids (D-arginine and D-ornithine metabolism, ko00472), metabolism of lipids (primary bile acid biosynthesis, ko00120; and secondary bile acid biosynthesis, ko00121), metabolism of terpenoids and polyketides (carotenoid biosynthesis, ko00906) and infectious disease (*Staphylococcus aureus* infection, ko05150) were upregulated in laboratory-reared triatomines (*p* < 0.05). In contrast, pathways such as those associated with glycan biosynthesis and metabolism (N-glycan biosynthesis, ko00510; glycosphingolipid biosynthesis - ganglio series, ko00604; and lipopolysaccharide biosynthesis, ko00540), carbohydrate metabolism (starch and sucrose metabolism, ko00500; and ascorbate and aldarate metabolism, ko00053), amino acid metabolism (glutathione metabolism, ko00480), lipid metabolism (alpha-linolenic acid metabolism, ko00592), terpenoid and polyketide metabolism (biosynthesis of siderophore group nonribosomal peptides, ko01053), the immune system (RIG-I-like receptor signaling pathway, ko04622; antigen processing and presentation, ko04612; and NOD-like receptor signaling pathway, ko04621), cell motility (flagellar assembly, ko02040; and bacterial chemotaxis, ko02030), membrane transport (bacterial secretion system, ko03070), and infectious disease (Chagas disease, ko05142; and African trypanosomiasis, ko05143) were enriched in wild-caught triatomines (*p* < 0.05).

## Discussion

Based on the adaptation of triatomines to human dwellings, these insects have traditionally been classified into four categories: sylvatic species, intrusive species, domiciliary species and domestic species (Carbajal-de-la-Fuente et al., 2019). *T. rubrofasciata*, as one kind of domiciliary species, has recently been frequently captured in human houses or near living areas in southern China (Liu et al., 2017; Huang et al., 2018; Hu et al., 2019; Shi et al., 2020), and residents who were reportedly bitten by this insect showed some clinical symptoms, including an urticaria-like systemic skin response or anaphylactic shock (Huang et al., 2018; Shi et al., 2020). Although *T. cruzi* has not been detected in wild-caught *T. rubrofasciata* in China to date, increased attention should be paid to the prevention and control of this vector due to the living habit of *T. rubrofasciata*. In addition, the feeding sources of wild-caught *T. rubrofasciata* were not identified in the present study, possibly because the triatomines that we caught were starved at that time, so there was limited blood in their guts, making it difficult to extract DNA and perform PCRs. Therefore, we could not draw the conclusion that these wild-caught triatomines did not ingest blood meals, because they have high resistance to starvation, and the volume of blood ingested by adults was proportionally lower than that ingested by nymphs (Cortéz and Gonçalves, 1998; Orantes et al., 2018; Hieu et al., 2019).

In this study, we demonstrated continuous dramatic changes in the gut microbes of *T. rubrofasciata* during development with *16S* rRNA sequencing. Diversity analyses revealed a relative increase in alpha diversity, whereas a relative reduction in beta diversity was observed during bug development, which indicated that the composition of the gut microbiota in *T. rubrofasciata* became increasingly similar with age, which is consistent with a previous study of *Triatoma sordida* (*T. sordida*) (Oliveira et al., 2018). Notably, the triatomines that we used for this part of the study were laboratory reared. Although the environmental conditions during feeding, such as temperature, humidity and blood meal sources, were all the same, the differences in the gut microbiome across developmental stages were still distinct, which indicated that some significant microbes were tightly linked to the development and growth of *T. rubrofasciata*, such as maturation of the immune system, selection of specific bacterial taxa, supplementation of nutrient or digestion (Oliveira et al., 2018).

The predominant phylum among the gut microbes of *T. rubrofasciata* was Firmicutes, which was consistent with the bacterial community in the salivary glands of *Rhodnius prolixus* (*R. prolixus*) (Lima et al., 2018). In addition, the abundance of this phylum increased markedly after the 1^st^ nymphal stage, and the same phenomenon was also observed in *T. sordida* (Oliveira et al., 2018). Conversely, the phylum Proteobacteria, which had the highest relative abundance in the salivary glands of *Triatoma brasiliensis*, *Triatoma infestans* (*T. infestans*), *Triatoma rubrovaria* and *Rhodnius milesi* (Lima et al., 2018), as well as in the gut of *Triatoma maculata* and *Rhodnius pallescens* (Montoya-Porras et al., 2018; Kieran et al., 2019), was highly depleted upon reaching the 2^nd^ nymphal stage of *T. rubrofasciata*, this result was opposite to that for *T. sordida* (Oliveira et al., 2018). Furthermore, the variance in gut bacteria between *T. rubrofasciata* and *T. sordida* was also reflected in the abundance of the phylum Actinobacteria, which increased significantly through the development of the former. The gut microbes found in different triatomines form a symbiotic relationship, and the presence of symbiotic organisms can affect the development and survival of both hosts and parasites (Garcia et al., 2010; de Fuentes-Vicente et al., 2018; Salcedo-Porras and Lowenberger, 2019). Among these symbionts, Actinobacteria can provide B complex vitamins, extracellular enzymes, secondary metabolites and antimicrobial bioactive compounds to the host, while Proteobacteria can inhibit the growth of pathogens transmitted by insect vectors (Gumiel et al., 2015; Oliveira et al., 2018). In addition, the results of LEfSe demonstrated that Proteobacteria was the main biomarker in the nymphal stage of *T. rubrofasciata*, whereas Firmicutes that produce antimicrobial molecules including polyketides and lipopeptides (Aleti et al., 2015) became dominant in the adult stage. Thus, further studies of the complex and dynamic gut microbes may be helpful in finding their potential effects on the host fitness.

Previous studies of the gut bacteria in *Triatoma dimidiata* showed that *Staphylococcus* was identified as the main bacterial genus (Dumonteil et al., 2018), and it was common to all the developmental stages of *T. sordida* but without obvious differences in abundance (Oliveira et al., 2018); however, our study found that *Staphylococcus* had a high relative abundance from the 1^st^ nymphal stage onwards. Interestingly, its abundance was associated with that of *Serratia*. Moreover, *Serratia* and *Enterobacter* were prevalent in the gut microbiome of *T. rubrofasciata*, and the result is consistent with that of a prior study of triatomines, including *T. infestans*, *Triatoma pseudomaculata*, *Panstrongylus megistus*, and *R. prolixus* (Azambuja et al., 2004). Both *Serratia* and *Enterobacter* are members of the family Enterobacteriaceae, which appears frequently in insect vectors, particularly in those whose diets are restricted to a few food sources, and Enterobacteriaceae may play an important role in host fitness by resisting pathogenic microbes. Some members of Enterobacteriaceae are able to kill closely related bacteria to reduce competition for essential nutrients (da Mota et al., 2012; da Mota et al., 2018; Montoya-Porras et al., 2018). Nevertheless, various studies have focused on *Serratia* because it is thought to decrease the population of *T. cruzi* by attacking the parasite’s membrane to impede the establishment of this pathogen (Flores-Villegas et al., 2015). The trypanocidal activity of *Serratia* was reported only under *in vitro* conditions, and its protective effect, preventing colonization by *T. cruzi in vivo*, was not distinct. It was hypothesized that the expression of bacterial cytotoxic genes varied among specific regions of the digestive tract (Gumiel et al., 2015; da Mota et al., 2018). Moreover, the diversity of *Serratia* species defines the influence of this genus on the pathogen or the host. While *Serratia* Y1 has the ability to inhibit the development of *Plasmodium berghei* in *Anopheles* by activating the mosquito immune system, *Serratia odorifera* enhances the susceptibility of *Aedes aegypti* to DENV-2 infection, and *Serratia marcescens* is pathogenic to honey bees (Raymann et al., 2018; Bai et al., 2019; Wu et al., 2019). Our results revealed that *Serratia* was enriched in 1^st^ stage nymphs, and as we could not artificially infect *T. rubrofasciata* with *T. cruzi*, the relationship between *Serratia* and *T. cruzi in vivo* was not clear. Likewise, *Enterobacter* was the common bacterium in the gut of *Aedes*, *Anopheles*, and *Culex*, and it could inhibit invasion by and the development of *Plasmodium falciparum via* the generation of reactive oxygen species (ROS) in *Anopheles* (Cirimotich et al., 2011; Jayakrishnan et al., 2018), but its impact on *T. cruzi* has not yet been studied. The abundance of *Enterobacter* in *T. rubrofasciata* did not change significantly, which suggested that this bacterium may play a vital role in the biology of the vector or in the transmission of pathogens. *Enterobacter* species have shown strong hemolytic activity and are capable of performing red blood cell lysis to accelerate blood meal digestion (Muturi et al., 2019). More importantly, the potential of *Enterobacter* and *Serratia* in the paratransgenic control of malaria has been reported widely (Eappen et al., 2013; Koosha et al., 2019a; Koosha et al., 2019b).

In other instances, diversity analyses between wild-caught and laboratory-reared *T. rubrofasciata* suggested that the gut bacterial populations increased dramatically in the latter, contributing to the ingested blood meal. Previous researches have shown that after blood ingestion, the number of gut bacteria increased rapidly, probably due to the richness of iron and protein in the blood meal (Azambuja et al., 2004; Castro et al., 2012). Hence, the restriction of blood feeding may contribute to the low diversity in the gut microbiota of wild-caught triatomines (Gumiel et al., 2015). Similarly, the PCoA plot suggested that the alterations in bacterial communities were also influenced by the bloodmeal of *T. rubrofasciata*, which was explained by the most significant phylum Proteobacteria, with the majority of these bacteria belonging to the class Gammaproteobacteria, while the differentiating genus was *Staphylococcus*, followed by *Enterococcus*, members of the class Bacilli, and the phylum Firmicutes.

Under laboratory conditions, *Staphylococcus* demonstrated a stronger colonization capacity in the gut of *T. rubrofasciata* than other natural bacteria, such as *Pantoea*, but the opposite result was detected in the gut flora of *Aedes albopictus* and *Culex quinquefasciatus* (Gazzoni Araújo Gonçalves et al., 2019). One of the possible reasons is that our wild-caught triatomines had not ingested blood. For the same reason, *Serratia* was not detected in the gut flora of field-caught *T. rubrofasciata*. Because *Staphylococcus* is a common constituent of the natural skin flora of animals, it would proliferate rapidly in the guts of triatomines after a blood meal. Notably, *Staphylococcus* is also an opportunistic pathogen; once people are bitten by *T. rubrofasciata*, secondary bacterial infection occurs, since the vector that transports pathogenic microbes could facilitate colonization by and multiplication of opportunistic invaders by providing an ideal environment. An analogous situation was observed in canid species infected with *Sarcoptes scabiei* mites (DeCandia et al., 2019). Therefore, based on the functional profile of gut bacterial communities predicted by the PICRUSt algorithm, we should pay more attention to the potential of interspecies transmission of the microbiota.

Under natural conditions, *Pantoea* and *Bacillus* were the dominant genera in the gut of *T. rubrofasciata*, which is opposite to the result of a previous study of *T. infestans* (Waltmann et al., 2019), and interestingly, *Pantoea* was absent in the gut bacteria of *T. rubrofasciata*. These phenomena indicated that these genera can be strongly influenced by environmental changes and the consequent immune responses of triatomines. Moreover, *Pantoea* and *Bacillus* are common in *Anopheles* as well as in natural and laboratory-reared sand flies; these bacteria are able to regulate the immune responses of sand fly larvae and could be used as paratransgenic tools against malaria or leishmaniasis (Dinparast Djadid et al., 2011; Akhoundi et al., 2012; Heerman et al., 2015; Karimian et al., 2019). Nevertheless, due to the limited number of natural *T. rubrofasciata* analyzed in the present study, the variations in the gut microbiome composition of wild populations across a wider geographic area and their correlation with *T. cruzi* infection will require further investigation.

In summary, we profiled the gut microbiome alterations of *T. rubrofasciata* across developmental stages, as well as its gut microbiota succession, under natural and laboratory conditions. Importantly, we observed significant differences in both the diversity and composition of the gut microbes of *T. rubrofasciata* at different ages and environmental statuses. The specific gut bacteria may be modulated by feeding type and may have an effect on *T. cruzi* infection; however, further exploration is essential for determining whether the microbiota changes identified are causal and identifying the important metabolic pathways of gut microbes using metagenomics. Moreover, understanding the interactions among vectors-*T. cruzi*-symbionts (including *Serratia*, *Enterobacter*, *Pantoea* and *Bacillus*), along with understanding the biological functions and potential antiparasitic activity *in vivo* of gut symbionts might lead to the application of these bacteria in paratransgenic control of American trypanosomiasis.

## Data Availability Statement

The sequencing data for the *16S* rRNA gene have been deposited in the NCBI Sequence Read Archive under project number PRJNA645287.

## Author Contributions

ZL, JY, and HX conceived and designed the experiments. ZL, YH, and HX drafted the manuscript. HX collected the samples. YH and MG performed the experiments. YH, MG, and PH collected and analyzed the data. HZ, YM, MZ, and JL participated in study design, technological guidance, and coordination. All authors contributed to the article and approved the submitted version.

## Funding

This work was supported by grants from the National Natural Science Foundation of China (grant no. 81572023 and 81371836), the National Parasitic Resources Center of China (grant no. NPRC-2019-194-30), Science and Technology Planning Project of Guangdong Province (grant no. 2019B030316025), Natural Science Foundation of Guangdong Province (grant no. 2019A1515011541), the National Key Research and Development Program of China (grant no. 2016YFC1202000 and 2016YFC1200500), the Open Foundation of Key Laboratory of Tropical Translational Medicine of Ministry of Education, Hainan Medical University (grant no. 2020TTM007), the 111 Project (grant no. B12003), Teaching Reform Project of Guangdong Province (grant no. 2017001) and Construction of Fujian Provincial Scientific and Technological Innovation Platform (2019Y2001).

## Conflict of Interest

The authors declare that the research was conducted in the absence of any commercial or financial relationships that could be construed as a potential conflict of interest.

## Footnotes

1
http://blast.ncbi.nlm.nih.gov/Blast.cgi

2
http://www.usadellab.org/cms/index.php?page=trimmomatic

3
https://github.com/torognes/vsearch

4
http://www.mothur.org

5
http://huttenhower.org/galaxy

6
http://picrust.github.com

7
http://greengenes.lbl.gov

8
http://www.genome.jp/kegg/pathway.html

## Supplementary Material

Supplementary Figure 1 Hierarchical clustering heatmap of PICRUSt analysis with annotated *16S* sequencing data demonstrates significant level 3 KEGG pathways between laboratory-reared and wild-caught *T. rubrofasciata*. Samples are shown in columns, and KEGG pathways are shown in rows.

Supplementary Table 1 OTU tables and taxonomic classifications of the *16S* rRNA gene.

Supplementary Table 2 Good’s coverage estimators of *T. rubrofasciata* gut samples (N1, 1^st^ stage nymphs; N2, 2^nd^ stage nymphs; N3, 3^rd^ stage nymphs; N4, 4^th^ stage nymphs; N5, 5^th^ stage nymphs; F, female adult; M, male adult).

Supplementary Table 3 One-way ANOVA of relative abundances of gut microbiome constituents across developmental stages of *T. rubrofasciata* at the phylum level (N1, 1^st^ stage nymphs; N2, 2^nd^ stage nymphs; N3, 3^rd^ stage nymphs; N4, 4^th^ stage nymphs; N5, 5^th^ stage nymphs; F, female adult; M, male adult).

Supplementary Table 4 One-way ANOVA of relative abundances of gut microbiome constituents across developmental stages of *T. rubrofasciata* at the genus level (N1, 1^st^ stage nymphs; N2, 2^nd^ stage nymphs; N3, 3^rd^ stage nymphs; N4, 4^th^ stage nymphs; N5, 5^th^ stage nymphs; F, female adult; M, male adult).

Supplementary Table 5 One-way ANOVA of relative abundances of gut microbiome constituents in laboratory-reared and wild-caught *T. rubrofasciata* at the phylum level.

Supplementary Table 6 One-way ANOVA of relative abundances of gut microbiome constituents in laboratory-reared and wild-caught *T. rubrofasciata* at the genus level.
