# Supplementary figures and images for: Dynamic of Composition and Diversity of Gut Microbiota in Triatoma rubrofasciata in Different Developmental Stages and Environmental Conditions
Source: Front Cell Infect Microbiol. 2020 Nov 2;10:587708. doi: 10.3389/fcimb.2020.587708 (PMC7667259; doi:10.3389/fcimb.2020.587708)

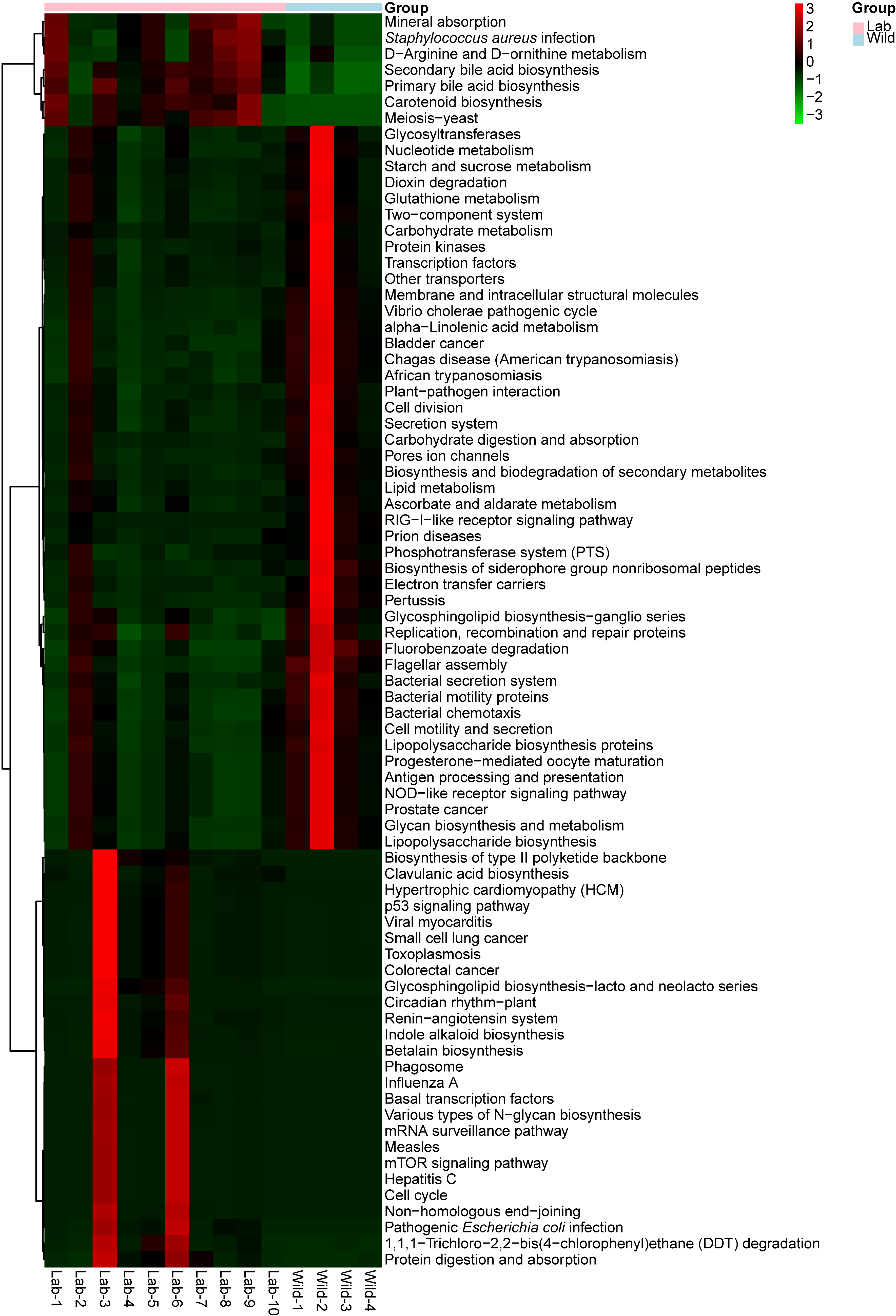

Supplement: Supplementary Figure 1 — Hierarchical clustering heatmap of PICRUSt analysis with annotated 16S sequencing data demonstrates significant level 3 KEGG pathways between laboratory-reared and wild-caught T. rubrofasciata. Samples are shown in columns, and KEGG pathways are shown in rows. [file Image_1.tif]
