# Supplementary material for: Dynamic of Composition and Diversity of Gut Microbiota in Triatoma rubrofasciata in Different Developmental Stages and Environmental Conditions
Source: Front Cell Infect Microbiol. 2020 Nov 2;10:587708. doi: 10.3389/fcimb.2020.587708 (PMC7667259; doi:10.3389/fcimb.2020.587708)
Supplement: Supplementary Table 1 — OTU tables and taxonomic classifications of the 16S rRNA gene. [file DataSheet_1.zip › Supplementary Table S2.DOCX]

| **Samples** | **Good's coverage (%)** |
| --- | --- |
| N1-1 | 99.75 |
| N1-2 | 99.77 |
| N1-3 | 99.68 |
| N1-4 | 99.24 |
| N1-5 | 99.64 |
| N1-6 | 99.84 |
| N1-7 | 99.76 |
| N2-1 | 99.49 |
| N2-2 | 99.67 |
| N2-3 | 99.79 |
| N2-4 | 99.61 |
| N2-5 | 98.83 |
| N2-6 | 99.69 |
| N2-7 | 99.65 |
| N3-1 | 98.76 |
| N3-2 | 99.20 |
| N3-3 | 99.48 |
| N3-4 | 98.76 |
| N3-5 | 99.29 |
| N3-6 | 99.42 |
| N3-7 | 99.60 |
| N4-1 | 99.41 |
| N4-2 | 98.84 |
| N4-3 | 99.96 |
| N4-4 | 99.67 |
| N4-5 | 99.70 |
| N4-6 | 99.73 |
| N4-7 | 99.62 |
| N5-1 | 99.85 |
| N5-2 | 99.90 |
| N5-3 | 99.79 |
| N5-4 | 99.84 |
| N5-5 | 99.68 |
| N5-6 | 99.62 |
| N5-7 | 99.37 |
| F-1 | 99.43 |
| F-2 | 99.85 |
| F-3 | 95.01 |
| F-4 | 98.72 |
| F-5 | 98.21 |
| M-1 | 96.37 |
| M-2 | 98.72 |
| M-3 | 99.12 |
| M-4 | 98.87 |
| M-5 | 99.25 |
| Wild-1 | 99.95 |
| Wild-2 | 99.98 |
| Wild-3 | 99.89 |
| Wild-4 | 99.63 |
