# Supplementary material for: Dynamic of Composition and Diversity of Gut Microbiota in Triatoma rubrofasciata in Different Developmental Stages and Environmental Conditions
Source: Front Cell Infect Microbiol. 2020 Nov 2;10:587708. doi: 10.3389/fcimb.2020.587708 (PMC7667259; doi:10.3389/fcimb.2020.587708)
Supplement: Supplementary Table 1 — OTU tables and taxonomic classifications of the 16S rRNA gene. [file DataSheet_1.zip › Supplementary Table S3.DOCX]

| **Phylum** | ***p*-value** | **FDR** | **N1** | **N2** | **N3** | **N4** | **N5** | **F** | **M** |
| --- | --- | --- | --- | --- | --- | --- | --- | --- | --- |
| Firmicutes | 0.00001 | 0.00018 | 0.0343 | 0.8468 | 0.9597 | 0.8532 | 0.8538 | 0.6615 | 0.6339 |
| Actinobacteria | 0.00057 | 0.00588 | 0.0013 | 0.0016 | 0.0010 | 0.0013 | 0.0016 | 0.0166 | 0.0526 |
| Proteobacteria | 0.00074 | 0.00588 | 0.8216 | 0.1254 | 0.0090 | 0.1380 | 0.1410 | 0.2177 | 0.1912 |
| Nitrospirae | 0.02790 | 0.16739 | 0.0000 | 0.0000 | 0.0000 | 0.0000 | 0.0000 | 0.0002 | 0.0004 |
| Other | 0.17565 | 0.37447 | 0.0000 | 0.0000 | 0.0001 | 0.0001 | 0.0001 | 0.0015 | 0.0008 |
| Thaumarchaeota | 0.18763 | 0.37447 | 0.0000 | 0.0000 | 0.0000 | 0.0000 | 0.0000 | 0.0027 | 0.0045 |
| Deinococcus-Thermus | 0.23712 | 0.37447 | 0.0000 | 0.0000 | 0.0000 | 0.0000 | 0.0000 | 0.0001 | 0.0000 |
| Chloroflexi | 0.23712 | 0.37447 | 0.0000 | 0.0000 | 0.0000 | 0.0000 | 0.0000 | 0.0001 | 0.0000 |
| Elusimicrobia | 0.23712 | 0.37447 | 0.0000 | 0.0000 | 0.0000 | 0.0000 | 0.0000 | 0.0001 | 0.0000 |
| Fusobacteria | 0.24889 | 0.37447 | 0.0000 | 0.0000 | 0.0003 | 0.0002 | 0.0000 | 0.0003 | 0.0008 |
| Patescibacteria | 0.26154 | 0.37447 | 0.0006 | 0.0000 | 0.0000 | 0.0000 | 0.0000 | 0.0000 | 0.0002 |
| Gemmatimonadetes | 0.26233 | 0.37447 | 0.0000 | 0.0000 | 0.0000 | 0.0000 | 0.0000 | 0.0001 | 0.0006 |
| Acidobacteria | 0.26502 | 0.37447 | 0.0000 | 0.0000 | 0.0000 | 0.0000 | 0.0000 | 0.0004 | 0.0005 |
| Tenericutes | 0.28029 | 0.37447 | 0.0012 | 0.0010 | 0.0002 | 0.0002 | 0.0002 | 0.0001 | 0.0002 |
| Euryarchaeota | 0.28470 | 0.37447 | 0.0000 | 0.0000 | 0.0000 | 0.0000 | 0.0000 | 0.0145 | 0.0185 |
| Bacteroidetes | 0.28955 | 0.37447 | 0.0317 | 0.0243 | 0.0288 | 0.0068 | 0.0029 | 0.0577 | 0.0698 |
| Crenarchaeota | 0.29170 | 0.37447 | 0.0000 | 0.0000 | 0.0000 | 0.0000 | 0.0000 | 0.0241 | 0.0231 |
| Diapherotrites | 0.30871 | 0.37447 | 0.0000 | 0.0000 | 0.0000 | 0.0000 | 0.0000 | 0.0003 | 0.0004 |
| Altiarchaeota | 0.30948 | 0.37447 | 0.0000 | 0.0000 | 0.0000 | 0.0000 | 0.0000 | 0.0005 | 0.0004 |
| Nanoarchaeaeota | 0.31205 | 0.37447 | 0.0000 | 0.0000 | 0.0000 | 0.0000 | 0.0000 | 0.0002 | 0.0002 |
| Epsilonbacteraeota | 0.39344 | 0.44964 | 0.0007 | 0.0002 | 0.0003 | 0.0001 | 0.0001 | 0.0011 | 0.0017 |
| Cyanobacteria | 0.50921 | 0.54598 | 0.1077 | 0.0003 | 0.0002 | 0.0000 | 0.0002 | 0.0003 | 0.0000 |
| Deferribacteres | 0.52323 | 0.54598 | 0.0008 | 0.0004 | 0.0002 | 0.0000 | 0.0001 | 0.0001 | 0.0000 |
| Spirochaetes | 0.65213 | 0.65213 | 0.0000 | 0.0000 | 0.0001 | 0.0000 | 0.0000 | 0.0001 | 0.0001 |
