# Supplementary material for: Dynamic of Composition and Diversity of Gut Microbiota in Triatoma rubrofasciata in Different Developmental Stages and Environmental Conditions
Source: Front Cell Infect Microbiol. 2020 Nov 2;10:587708. doi: 10.3389/fcimb.2020.587708 (PMC7667259; doi:10.3389/fcimb.2020.587708)
Supplement: Supplementary Table 1 — OTU tables and taxonomic classifications of the 16S rRNA gene. [file DataSheet_1.zip › Supplementary Table S4.DOCX]

| **Genus** | ***p*-value** | **FDR** | **N1** | **N2** | **N3** | **N4** | **N5** | **F** | **M** |
| --- | --- | --- | --- | --- | --- | --- | --- | --- | --- |
| *Staphylococcus* | 0.00001 | 0.00351 | 0.0007 | 0.8313 | 0.9420 | 0.8489 | 0.8511 | 0.5679 | 0.5471 |
| *MND1* | 0.00087 | 0.11811 | 0.0000 | 0.0000 | 0.0000 | 0.0000 | 0.0000 | 0.0002 | 0.0000 |
| *Serratia* | 0.00124 | 0.11811 | 0.4968 | 0.0016 | 0.0008 | 0.0017 | 0.0013 | 0.0001 | 0.1786 |
| *metagenome* | 0.00330 | 0.19295 | 0.0000 | 0.0000 | 0.0001 | 0.0000 | 0.0000 | 0.0000 | 0.0000 |
| *Sphingomonas* | 0.00339 | 0.19295 | 0.0000 | 0.0001 | 0.0000 | 0.0000 | 0.0000 | 0.0011 | 0.0001 |
| *Altererythrobacter* | 0.00741 | 0.30273 | 0.0003 | 0.0012 | 0.0009 | 0.0006 | 0.0000 | 0.0000 | 0.0000 |
| *Christensenellaceae R-7 group* | 0.01028 | 0.30273 | 0.0000 | 0.0000 | 0.0001 | 0.0000 | 0.0000 | 0.0006 | 0.0014 |
| *Burkholderia-Caballeronia-Paraburkholderia* | 0.01139 | 0.30273 | 0.3151 | 0.0016 | 0.0001 | 0.0001 | 0.0001 | 0.0000 | 0.0000 |
| *Ruminococcaceae UCG-014* | 0.01227 | 0.30273 | 0.0001 | 0.0003 | 0.0003 | 0.0001 | 0.0001 | 0.0003 | 0.0058 |
| *Senegalimassilia* | 0.01283 | 0.30273 | 0.0000 | 0.0000 | 0.0000 | 0.0000 | 0.0000 | 0.0000 | 0.0002 |
| *Lysinibacillus* | 0.01283 | 0.30273 | 0.0000 | 0.0000 | 0.0000 | 0.0000 | 0.0000 | 0.0002 | 0.0000 |
| *Pseudarthrobacter* | 0.01387 | 0.30273 | 0.0001 | 0.0000 | 0.0000 | 0.0000 | 0.0000 | 0.0005 | 0.0004 |
| *Atopostipes* | 0.01419 | 0.30273 | 0.0000 | 0.0000 | 0.0000 | 0.0000 | 0.0000 | 0.0000 | 0.0003 |
| *Turicibacter* | 0.01487 | 0.30273 | 0.0000 | 0.0000 | 0.0003 | 0.0000 | 0.0000 | 0.0005 | 0.0012 |
| *[Eubacterium] xylanophilum group* | 0.01821 | 0.34593 | 0.0002 | 0.0001 | 0.0001 | 0.0000 | 0.0000 | 0.0001 | 0.0008 |
| *Lachnospiraceae UCG-010* | 0.02082 | 0.34929 | 0.0000 | 0.0000 | 0.0000 | 0.0000 | 0.0000 | 0.0004 | 0.0000 |
| *Lactobacillus* | 0.02084 | 0.34929 | 0.0002 | 0.0000 | 0.0005 | 0.0002 | 0.0003 | 0.0041 | 0.0043 |
| *Odoribacter* | 0.02303 | 0.36465 | 0.0023 | 0.0050 | 0.0012 | 0.0006 | 0.0002 | 0.0008 | 0.0022 |
| *Intestinimonas* | 0.02801 | 0.42016 | 0.0001 | 0.0000 | 0.0000 | 0.0000 | 0.0000 | 0.0000 | 0.0004 |
| *Psychroglaciecola* | 0.03024 | 0.43093 | 0.0000 | 0.0000 | 0.0000 | 0.0000 | 0.0000 | 0.0000 | 0.0002 |
| *Prevotellaceae NK3B31 group* | 0.03333 | 0.43275 | 0.0005 | 0.0000 | 0.0001 | 0.0002 | 0.0000 | 0.0004 | 0.0021 |
| *Ruminiclostridium 6* | 0.03341 | 0.43275 | 0.0000 | 0.0000 | 0.0001 | 0.0000 | 0.0000 | 0.0002 | 0.0004 |
| *Oceanicaulis* | 0.03505 | 0.43431 | 0.0013 | 0.0054 | 0.0032 | 0.0025 | 0.0010 | 0.0000 | 0.0000 |
| *Fusicatenibacter* | 0.03800 | 0.45124 | 0.0001 | 0.0000 | 0.0000 | 0.0000 | 0.0000 | 0.0005 | 0.0009 |
| *Ruminococcaceae UCG-005* | 0.04299 | 0.49003 | 0.0000 | 0.0000 | 0.0001 | 0.0000 | 0.0000 | 0.0016 | 0.0024 |
| *Desulfovibrio* | 0.04850 | 0.51180 | 0.0004 | 0.0000 | 0.0003 | 0.0000 | 0.0002 | 0.0005 | 0.0015 |
| *uncultured organism* | 0.04922 | 0.51180 | 0.0000 | 0.0000 | 0.0002 | 0.0000 | 0.0000 | 0.0004 | 0.0015 |
| *Rhodanobacter* | 0.05028 | 0.51180 | 0.0000 | 0.0000 | 0.0000 | 0.0000 | 0.0000 | 0.0000 | 0.0003 |
| *Rickettsia* | 0.05548 | 0.52625 | 0.0000 | 0.0000 | 0.0000 | 0.0000 | 0.0000 | 0.0001 | 0.0000 |
| *Ellin6067* | 0.05578 | 0.52625 | 0.0000 | 0.0000 | 0.0000 | 0.0000 | 0.0000 | 0.0001 | 0.0008 |
| *Ambiguous taxa* | 0.06381 | 0.52625 | 0.0010 | 0.0004 | 0.0008 | 0.0005 | 0.0002 | 0.2013 | 0.0016 |
| *Asinibacterium* | 0.07109 | 0.52625 | 0.0000 | 0.0000 | 0.0000 | 0.0000 | 0.0001 | 0.0000 | 0.0000 |
| *Ruminococcaceae UCG-013* | 0.07493 | 0.52625 | 0.0000 | 0.0000 | 0.0000 | 0.0000 | 0.0000 | 0.0001 | 0.0006 |
| *Haemophilus* | 0.07580 | 0.52625 | 0.0000 | 0.0000 | 0.0002 | 0.0000 | 0.0000 | 0.0001 | 0.0000 |
| *Phenylobacterium* | 0.08200 | 0.52625 | 0.0000 | 0.0000 | 0.0000 | 0.0000 | 0.0000 | 0.0001 | 0.0002 |
| *Ruminococcaceae UCG-010* | 0.08404 | 0.52625 | 0.0000 | 0.0000 | 0.0000 | 0.0000 | 0.0000 | 0.0001 | 0.0003 |
| *Parviterribacter* | 0.08415 | 0.52625 | 0.0000 | 0.0000 | 0.0000 | 0.0000 | 0.0000 | 0.0006 | 0.0003 |
| *Jeotgalicoccus* | 0.09037 | 0.52625 | 0.0000 | 0.0000 | 0.0000 | 0.0000 | 0.0000 | 0.0008 | 0.0003 |
| *Nocardiopsis* | 0.09188 | 0.52625 | 0.0000 | 0.0006 | 0.0000 | 0.0000 | 0.0000 | 0.0003 | 0.0001 |
| *Ruminococcus 2* | 0.09294 | 0.52625 | 0.0001 | 0.0001 | 0.0003 | 0.0000 | 0.0000 | 0.0020 | 0.0009 |
| *Bradyrhizobium* | 0.09394 | 0.52625 | 0.0000 | 0.0000 | 0.0000 | 0.0000 | 0.0000 | 0.0003 | 0.0001 |
| *Neisseria* | 0.09695 | 0.52625 | 0.0000 | 0.0000 | 0.0003 | 0.0001 | 0.0000 | 0.0000 | 0.0000 |
| *Caulobacter* | 0.09778 | 0.52625 | 0.0000 | 0.0000 | 0.0000 | 0.0000 | 0.0000 | 0.0003 | 0.0002 |
| *Glycomyces* | 0.09917 | 0.52625 | 0.0000 | 0.0000 | 0.0000 | 0.0000 | 0.0000 | 0.0001 | 0.0004 |
| *Arthrobacter* | 0.11301 | 0.52625 | 0.0000 | 0.0000 | 0.0000 | 0.0000 | 0.0000 | 0.0004 | 0.0003 |
| *Ruminococcaceae NK4A214 group* | 0.11470 | 0.52625 | 0.0000 | 0.0000 | 0.0000 | 0.0000 | 0.0000 | 0.0012 | 0.0017 |
| *Clostridium sensu stricto 1* | 0.11713 | 0.52625 | 0.0000 | 0.0002 | 0.0000 | 0.0000 | 0.0000 | 0.0013 | 0.0022 |
| *[Eubacterium] coprostanoligenes group* | 0.12300 | 0.52625 | 0.0002 | 0.0001 | 0.0002 | 0.0001 | 0.0000 | 0.0081 | 0.0058 |
| *Enhydrobacter* | 0.12951 | 0.52625 | 0.0000 | 0.0001 | 0.0000 | 0.0000 | 0.0000 | 0.0006 | 0.0003 |
| *Barnesiella* | 0.13211 | 0.52625 | 0.0000 | 0.0000 | 0.0000 | 0.0000 | 0.0000 | 0.0008 | 0.0003 |
| *Parasutterella* | 0.13283 | 0.52625 | 0.0000 | 0.0000 | 0.0000 | 0.0001 | 0.0000 | 0.0002 | 0.0008 |
| *Candidatus Arthromitus* | 0.13781 | 0.52625 | 0.0000 | 0.0000 | 0.0001 | 0.0000 | 0.0000 | 0.0001 | 0.0000 |
| *Massilia* | 0.14189 | 0.52625 | 0.0001 | 0.0000 | 0.0000 | 0.0000 | 0.0001 | 0.0005 | 0.0003 |
| *Escherichia-Shigella* | 0.14632 | 0.52625 | 0.0002 | 0.0001 | 0.0001 | 0.0001 | 0.0001 | 0.0054 | 0.0022 |
| *Romboutsia* | 0.14844 | 0.52625 | 0.0000 | 0.0000 | 0.0002 | 0.0000 | 0.0000 | 0.0033 | 0.0034 |
| *Faecalibacterium* | 0.14919 | 0.52625 | 0.0005 | 0.0002 | 0.0015 | 0.0002 | 0.0000 | 0.0046 | 0.0057 |
| *Ruminococcus 1* | 0.14980 | 0.52625 | 0.0001 | 0.0000 | 0.0001 | 0.0001 | 0.0000 | 0.0019 | 0.0017 |
| *Lachnoclostridium* | 0.15451 | 0.52625 | 0.0020 | 0.0003 | 0.0002 | 0.0000 | 0.0001 | 0.0005 | 0.0008 |
| *Corynebacterium 1* | 0.15852 | 0.52625 | 0.0000 | 0.0001 | 0.0001 | 0.0000 | 0.0000 | 0.0003 | 0.0001 |
| *[Ruminococcus] torques group* | 0.16469 | 0.52625 | 0.0001 | 0.0000 | 0.0002 | 0.0000 | 0.0000 | 0.0040 | 0.0032 |
| *Subdoligranulum* | 0.17030 | 0.52625 | 0.0000 | 0.0000 | 0.0001 | 0.0001 | 0.0000 | 0.0022 | 0.0034 |
| *uncultured bacterium* | 0.17030 | 0.52625 | 0.0096 | 0.0072 | 0.0048 | 0.0013 | 0.0009 | 0.0050 | 0.0177 |
| *Ruminococcaceae UCG-002* | 0.17376 | 0.52625 | 0.0001 | 0.0000 | 0.0001 | 0.0000 | 0.0000 | 0.0075 | 0.0031 |
| *Oscillibacter* | 0.17727 | 0.52625 | 0.0003 | 0.0000 | 0.0001 | 0.0000 | 0.0001 | 0.0002 | 0.0005 |
| *Alloprevotella* | 0.17786 | 0.52625 | 0.0012 | 0.0018 | 0.0009 | 0.0001 | 0.0000 | 0.0025 | 0.0040 |
| *Sneathia* | 0.19127 | 0.52625 | 0.0000 | 0.0000 | 0.0000 | 0.0000 | 0.0000 | 0.0001 | 0.0002 |
| *Family XIII AD3011 group* | 0.19127 | 0.52625 | 0.0000 | 0.0000 | 0.0000 | 0.0000 | 0.0000 | 0.0002 | 0.0001 |
| *Mycoplasma* | 0.19142 | 0.52625 | 0.0005 | 0.0006 | 0.0002 | 0.0001 | 0.0002 | 0.0000 | 0.0000 |
| *Eisenbergiella* | 0.19486 | 0.52625 | 0.0000 | 0.0000 | 0.0000 | 0.0000 | 0.0000 | 0.0030 | 0.0006 |
| *Erysipelotrichaceae UCG-003* | 0.19550 | 0.52625 | 0.0000 | 0.0000 | 0.0000 | 0.0000 | 0.0000 | 0.0005 | 0.0005 |
| *Gaiella* | 0.19904 | 0.52625 | 0.0000 | 0.0000 | 0.0000 | 0.0000 | 0.0000 | 0.0012 | 0.0009 |
| *Dorea* | 0.19971 | 0.52625 | 0.0000 | 0.0000 | 0.0001 | 0.0000 | 0.0000 | 0.0026 | 0.0009 |
| *Ruminococcaceae UCG-003* | 0.20115 | 0.52625 | 0.0001 | 0.0000 | 0.0000 | 0.0000 | 0.0000 | 0.0001 | 0.0002 |
| *Henriciella* | 0.20115 | 0.52625 | 0.0000 | 0.0000 | 0.0000 | 0.0002 | 0.0000 | 0.0000 | 0.0000 |
| *endosymbionts8* | 0.20367 | 0.52625 | 0.0000 | 0.0000 | 0.0000 | 0.0000 | 0.0000 | 0.0003 | 0.0004 |
| *Candidatus Nitrososphaera* | 0.20393 | 0.52625 | 0.0000 | 0.0000 | 0.0000 | 0.0000 | 0.0000 | 0.0003 | 0.0005 |
| *Acinetobacter* | 0.20460 | 0.52625 | 0.0003 | 0.0000 | 0.0002 | 0.0000 | 0.0000 | 0.0024 | 0.0010 |
| *Fusobacterium* | 0.20573 | 0.52625 | 0.0000 | 0.0000 | 0.0003 | 0.0001 | 0.0000 | 0.0002 | 0.0005 |
| *Hungatella* | 0.20633 | 0.52625 | 0.0000 | 0.0000 | 0.0000 | 0.0000 | 0.0000 | 0.0018 | 0.0005 |
| *Tyzzerella* | 0.20896 | 0.52625 | 0.0000 | 0.0001 | 0.0000 | 0.0000 | 0.0000 | 0.0000 | 0.0000 |
| *Butyricimonas* | 0.21432 | 0.52625 | 0.0000 | 0.0000 | 0.0000 | 0.0000 | 0.0000 | 0.0022 | 0.0010 |
| *Alistipes* | 0.21805 | 0.52625 | 0.0017 | 0.0023 | 0.0017 | 0.0002 | 0.0003 | 0.0079 | 0.0063 |
| *Lachnospira* | 0.21820 | 0.52625 | 0.0002 | 0.0000 | 0.0003 | 0.0000 | 0.0000 | 0.0000 | 0.0002 |
| *Prevotella 1* | 0.22277 | 0.52625 | 0.0000 | 0.0000 | 0.0001 | 0.0000 | 0.0000 | 0.0001 | 0.0003 |
| *[Clostridium] innocuum group* | 0.23102 | 0.52625 | 0.0000 | 0.0000 | 0.0000 | 0.0000 | 0.0000 | 0.0020 | 0.0004 |
| *Methanosarcina* | 0.23179 | 0.52625 | 0.0000 | 0.0000 | 0.0000 | 0.0000 | 0.0000 | 0.0012 | 0.0009 |
| *Hoyosella* | 0.23712 | 0.52625 | 0.0000 | 0.0000 | 0.0000 | 0.0000 | 0.0000 | 0.0000 | 0.0063 |
| *Faecalibaculum* | 0.23712 | 0.52625 | 0.0000 | 0.0000 | 0.0000 | 0.0000 | 0.0000 | 0.0012 | 0.0000 |
| *Candidatus Alysiosphaera* | 0.23712 | 0.52625 | 0.0000 | 0.0000 | 0.0000 | 0.0000 | 0.0000 | 0.0004 | 0.0000 |
| *Sphingobacterium* | 0.23712 | 0.52625 | 0.0000 | 0.0000 | 0.0000 | 0.0000 | 0.0000 | 0.0003 | 0.0000 |
| *Hymenobacter* | 0.23712 | 0.52625 | 0.0000 | 0.0000 | 0.0000 | 0.0000 | 0.0000 | 0.0000 | 0.0003 |
| *Actinoplanes* | 0.23712 | 0.52625 | 0.0000 | 0.0000 | 0.0000 | 0.0000 | 0.0000 | 0.0003 | 0.0000 |
| *Crossiella* | 0.23712 | 0.52625 | 0.0000 | 0.0000 | 0.0000 | 0.0000 | 0.0000 | 0.0000 | 0.0003 |
| *Prevotellaceae Ga6A1 group* | 0.23712 | 0.52625 | 0.0000 | 0.0000 | 0.0000 | 0.0000 | 0.0000 | 0.0002 | 0.0000 |
| *Clostridium sensu stricto 12* | 0.23712 | 0.52625 | 0.0000 | 0.0000 | 0.0000 | 0.0000 | 0.0000 | 0.0002 | 0.0000 |
| *mouse gut metagenome* | 0.23712 | 0.52625 | 0.0000 | 0.0000 | 0.0000 | 0.0000 | 0.0000 | 0.0002 | 0.0000 |
| *Granulicatella* | 0.23712 | 0.52625 | 0.0000 | 0.0000 | 0.0000 | 0.0000 | 0.0000 | 0.0002 | 0.0000 |
| *Flavonifractor* | 0.23712 | 0.52625 | 0.0000 | 0.0000 | 0.0000 | 0.0000 | 0.0000 | 0.0000 | 0.0002 |
| *Acetobacter* | 0.23712 | 0.52625 | 0.0000 | 0.0000 | 0.0000 | 0.0000 | 0.0000 | 0.0000 | 0.0002 |
| *Micromonospora* | 0.23712 | 0.52625 | 0.0000 | 0.0000 | 0.0000 | 0.0000 | 0.0000 | 0.0000 | 0.0001 |
| *Methyloversatilis* | 0.23712 | 0.52625 | 0.0000 | 0.0000 | 0.0000 | 0.0000 | 0.0000 | 0.0000 | 0.0001 |
| *Cytophaga* | 0.23712 | 0.52625 | 0.0000 | 0.0000 | 0.0000 | 0.0000 | 0.0000 | 0.0001 | 0.0000 |
| *Eggerthella* | 0.23712 | 0.52625 | 0.0000 | 0.0000 | 0.0000 | 0.0000 | 0.0000 | 0.0001 | 0.0000 |
| *Deinococcus* | 0.23712 | 0.52625 | 0.0000 | 0.0000 | 0.0000 | 0.0000 | 0.0000 | 0.0001 | 0.0000 |
| *GCA-900066755* | 0.23712 | 0.52625 | 0.0000 | 0.0000 | 0.0000 | 0.0000 | 0.0000 | 0.0001 | 0.0000 |
| *Fluviicola* | 0.23712 | 0.52625 | 0.0000 | 0.0000 | 0.0000 | 0.0000 | 0.0000 | 0.0001 | 0.0000 |
| *Nannocystis* | 0.23712 | 0.52625 | 0.0000 | 0.0000 | 0.0000 | 0.0000 | 0.0000 | 0.0001 | 0.0000 |
| *SWB02* | 0.23712 | 0.52625 | 0.0000 | 0.0000 | 0.0000 | 0.0000 | 0.0000 | 0.0001 | 0.0000 |
| *Dietzia* | 0.23712 | 0.52625 | 0.0000 | 0.0000 | 0.0000 | 0.0000 | 0.0000 | 0.0000 | 0.0001 |
| *Cetobacterium* | 0.23712 | 0.52625 | 0.0000 | 0.0000 | 0.0000 | 0.0000 | 0.0000 | 0.0000 | 0.0001 |
| *Pygmaiobacter* | 0.23712 | 0.52625 | 0.0000 | 0.0000 | 0.0000 | 0.0000 | 0.0000 | 0.0000 | 0.0001 |
| *Clade Ia* | 0.23712 | 0.52625 | 0.0000 | 0.0000 | 0.0000 | 0.0000 | 0.0000 | 0.0000 | 0.0001 |
| *Reyranella* | 0.23712 | 0.52625 | 0.0000 | 0.0000 | 0.0000 | 0.0000 | 0.0000 | 0.0000 | 0.0001 |
| *Terrimonas* | 0.23712 | 0.52625 | 0.0000 | 0.0000 | 0.0000 | 0.0000 | 0.0000 | 0.0001 | 0.0000 |
| *Faecalitalea* | 0.24131 | 0.52625 | 0.0000 | 0.0000 | 0.0000 | 0.0000 | 0.0000 | 0.0009 | 0.0005 |
| *Prevotellaceae UCG-003* | 0.24246 | 0.52625 | 0.0000 | 0.0000 | 0.0000 | 0.0000 | 0.0000 | 0.0001 | 0.0013 |
| *Allobaculum* | 0.24436 | 0.52625 | 0.0000 | 0.0000 | 0.0000 | 0.0000 | 0.0000 | 0.0002 | 0.0009 |
| *Comamonas* | 0.24610 | 0.52625 | 0.0002 | 0.0000 | 0.0000 | 0.0000 | 0.0000 | 0.0000 | 0.0000 |
| *Enteractinococcus* | 0.24708 | 0.52625 | 0.0000 | 0.0000 | 0.0001 | 0.0000 | 0.0000 | 0.0013 | 0.0000 |
| *Blautia* | 0.24814 | 0.52625 | 0.0003 | 0.0003 | 0.0004 | 0.0000 | 0.0000 | 0.0046 | 0.0025 |
| *Dialister* | 0.24846 | 0.52625 | 0.0000 | 0.0000 | 0.0001 | 0.0000 | 0.0000 | 0.0010 | 0.0004 |
| *Negativibacillus* | 0.25474 | 0.52625 | 0.0000 | 0.0000 | 0.0000 | 0.0000 | 0.0000 | 0.0005 | 0.0000 |
| *Brevundimonas* | 0.25657 | 0.52625 | 0.0001 | 0.0000 | 0.0000 | 0.0000 | 0.0000 | 0.0017 | 0.0000 |
| *Bacteroides* | 0.25691 | 0.52625 | 0.0065 | 0.0030 | 0.0094 | 0.0020 | 0.0007 | 0.0283 | 0.0157 |
| *Methanocella* | 0.25716 | 0.52625 | 0.0000 | 0.0000 | 0.0000 | 0.0000 | 0.0000 | 0.0007 | 0.0009 |
| *uncultured archaeon* | 0.25866 | 0.52625 | 0.0000 | 0.0000 | 0.0000 | 0.0000 | 0.0000 | 0.0008 | 0.0007 |
| *Collinsella* | 0.26063 | 0.52625 | 0.0001 | 0.0001 | 0.0001 | 0.0000 | 0.0000 | 0.0020 | 0.0018 |
| *Rice Cluster I* | 0.26421 | 0.52625 | 0.0000 | 0.0000 | 0.0000 | 0.0000 | 0.0000 | 0.0009 | 0.0013 |
| *Methanomassiliicoccus* | 0.26604 | 0.52625 | 0.0000 | 0.0000 | 0.0000 | 0.0000 | 0.0000 | 0.0011 | 0.0017 |
| *Adlercreutzia* | 0.26617 | 0.52625 | 0.0000 | 0.0000 | 0.0000 | 0.0000 | 0.0000 | 0.0060 | 0.0019 |
| *Roseburia* | 0.26892 | 0.52625 | 0.0003 | 0.0002 | 0.0009 | 0.0000 | 0.0001 | 0.0038 | 0.0032 |
| *Bifidobacterium* | 0.27061 | 0.52625 | 0.0002 | 0.0001 | 0.0002 | 0.0000 | 0.0001 | 0.0006 | 0.0016 |
| *Candidatus Nitrosotalea* | 0.27239 | 0.52625 | 0.0000 | 0.0000 | 0.0000 | 0.0000 | 0.0000 | 0.0008 | 0.0010 |
| *Vibrio* | 0.27266 | 0.52625 | 0.0000 | 0.0000 | 0.0000 | 0.0000 | 0.0000 | 0.0000 | 0.0003 |
| *Mesorhizobium* | 0.27266 | 0.52625 | 0.0000 | 0.0000 | 0.0000 | 0.0000 | 0.0000 | 0.0003 | 0.0000 |
| *Coprobacillus* | 0.27293 | 0.52625 | 0.0000 | 0.0000 | 0.0000 | 0.0000 | 0.0000 | 0.0018 | 0.0005 |
| *[Eubacterium] hallii group* | 0.27350 | 0.52625 | 0.0000 | 0.0000 | 0.0001 | 0.0000 | 0.0000 | 0.0030 | 0.0009 |
| *Roseitalea* | 0.27744 | 0.52625 | 0.0000 | 0.0003 | 0.0001 | 0.0001 | 0.0001 | 0.0000 | 0.0000 |
| *Candidatus Methanoperedens* | 0.27771 | 0.52625 | 0.0000 | 0.0000 | 0.0000 | 0.0000 | 0.0000 | 0.0009 | 0.0011 |
| *Muribaculum* | 0.27960 | 0.52625 | 0.0002 | 0.0002 | 0.0001 | 0.0000 | 0.0000 | 0.0002 | 0.0008 |
| *[Eubacterium] nodatum group* | 0.27993 | 0.52625 | 0.0000 | 0.0000 | 0.0000 | 0.0000 | 0.0000 | 0.0002 | 0.0001 |
| *[Eubacterium] ventriosum group* | 0.28160 | 0.52625 | 0.0000 | 0.0000 | 0.0000 | 0.0000 | 0.0000 | 0.0001 | 0.0003 |
| *Enterococcus* | 0.28229 | 0.52625 | 0.0000 | 0.0000 | 0.0001 | 0.0001 | 0.0000 | 0.0046 | 0.0018 |
| *Prevotella 7* | 0.28341 | 0.52625 | 0.0000 | 0.0000 | 0.0004 | 0.0002 | 0.0000 | 0.0000 | 0.0003 |
| *Candidatus Saccharimonas* | 0.28399 | 0.52625 | 0.0000 | 0.0000 | 0.0000 | 0.0000 | 0.0000 | 0.0000 | 0.0002 |
| *UBA1819* | 0.28399 | 0.52625 | 0.0000 | 0.0000 | 0.0000 | 0.0000 | 0.0000 | 0.0002 | 0.0000 |
| *Olsenella* | 0.28399 | 0.52625 | 0.0000 | 0.0000 | 0.0000 | 0.0000 | 0.0000 | 0.0000 | 0.0002 |
| *Coprococcus 1* | 0.28687 | 0.52625 | 0.0000 | 0.0000 | 0.0000 | 0.0000 | 0.0000 | 0.0003 | 0.0001 |
| *Gordonibacter* | 0.28687 | 0.52625 | 0.0000 | 0.0000 | 0.0000 | 0.0000 | 0.0000 | 0.0001 | 0.0003 |
| *uncultured methanogenic archaeon* | 0.29141 | 0.52625 | 0.0000 | 0.0000 | 0.0000 | 0.0000 | 0.0000 | 0.0203 | 0.0189 |
| *Coprococcus 3* | 0.29549 | 0.52625 | 0.0000 | 0.0000 | 0.0000 | 0.0000 | 0.0000 | 0.0004 | 0.0001 |
| *Ruminococcaceae UCG-004* | 0.29562 | 0.52625 | 0.0000 | 0.0000 | 0.0000 | 0.0000 | 0.0000 | 0.0005 | 0.0003 |
| *Candidatus Solibacter* | 0.29562 | 0.52625 | 0.0000 | 0.0000 | 0.0000 | 0.0000 | 0.0000 | 0.0001 | 0.0001 |
| *CL500-29 marine group* | 0.29562 | 0.52625 | 0.0000 | 0.0000 | 0.0000 | 0.0000 | 0.0000 | 0.0001 | 0.0001 |
| *Sanguibacteroides* | 0.29562 | 0.52625 | 0.0000 | 0.0000 | 0.0000 | 0.0000 | 0.0000 | 0.0001 | 0.0001 |
| *Ohtaekwangia* | 0.29562 | 0.52625 | 0.0000 | 0.0000 | 0.0000 | 0.0000 | 0.0000 | 0.0001 | 0.0001 |
| *Oceanobacillus* | 0.29562 | 0.52625 | 0.0000 | 0.0000 | 0.0000 | 0.0000 | 0.0000 | 0.0001 | 0.0001 |
| *Methanobacterium* | 0.29688 | 0.52625 | 0.0000 | 0.0000 | 0.0000 | 0.0000 | 0.0000 | 0.0080 | 0.0098 |
| *Methanosaeta* | 0.29958 | 0.52625 | 0.0000 | 0.0000 | 0.0000 | 0.0000 | 0.0000 | 0.0011 | 0.0019 |
| *GCA-900066575* | 0.30438 | 0.52625 | 0.0000 | 0.0000 | 0.0000 | 0.0000 | 0.0000 | 0.0000 | 0.0002 |
| *Bryobacter* | 0.30564 | 0.52625 | 0.0000 | 0.0000 | 0.0000 | 0.0000 | 0.0000 | 0.0001 | 0.0002 |
| *Motilibacter* | 0.30564 | 0.52625 | 0.0000 | 0.0000 | 0.0000 | 0.0000 | 0.0000 | 0.0002 | 0.0001 |
| *Pseudomonas* | 0.30605 | 0.52625 | 0.0003 | 0.0000 | 0.0000 | 0.0000 | 0.0002 | 0.0006 | 0.0000 |
| *uncultured Thermoplasmata archaeon* | 0.30754 | 0.52625 | 0.0000 | 0.0000 | 0.0000 | 0.0000 | 0.0000 | 0.0004 | 0.0003 |
| *Phascolarctobacterium* | 0.30871 | 0.52625 | 0.0000 | 0.0000 | 0.0000 | 0.0000 | 0.0000 | 0.0002 | 0.0002 |
| *Prevotella 2* | 0.31059 | 0.52625 | 0.0000 | 0.0000 | 0.0002 | 0.0000 | 0.0000 | 0.0010 | 0.0003 |
| *uncultured crenarchaeote* | 0.31205 | 0.52625 | 0.0000 | 0.0000 | 0.0000 | 0.0000 | 0.0000 | 0.0007 | 0.0007 |
| *Ferruginibacter* | 0.31205 | 0.52625 | 0.0000 | 0.0000 | 0.0000 | 0.0000 | 0.0000 | 0.0001 | 0.0001 |
| *Sphingobium* | 0.31205 | 0.52625 | 0.0000 | 0.0000 | 0.0000 | 0.0000 | 0.0000 | 0.0001 | 0.0001 |
| *uncultured Bacteroidetes bacterium* | 0.32161 | 0.53690 | 0.0001 | 0.0000 | 0.0000 | 0.0000 | 0.0000 | 0.0000 | 0.0003 |
| *Holdemanella* | 0.32214 | 0.53690 | 0.0000 | 0.0000 | 0.0000 | 0.0000 | 0.0000 | 0.0009 | 0.0004 |
| *uncultured* | 0.32676 | 0.54143 | 0.0060 | 0.0024 | 0.0020 | 0.0006 | 0.0005 | 0.0024 | 0.0048 |
| *Marvinbryantia* | 0.33787 | 0.55661 | 0.0001 | 0.0000 | 0.0002 | 0.0000 | 0.0000 | 0.0007 | 0.0005 |
| *uncultured gamma proteobacterium* | 0.34799 | 0.56351 | 0.0000 | 0.0000 | 0.0000 | 0.0000 | 0.0000 | 0.0000 | 0.0001 |
| *Actinomyces* | 0.34799 | 0.56351 | 0.0000 | 0.0000 | 0.0000 | 0.0000 | 0.0000 | 0.0001 | 0.0000 |
| *Nitrospira* | 0.34799 | 0.56351 | 0.0000 | 0.0000 | 0.0000 | 0.0000 | 0.0000 | 0.0001 | 0.0000 |
| *Anaerotruncus* | 0.35070 | 0.56469 | 0.0000 | 0.0000 | 0.0001 | 0.0000 | 0.0000 | 0.0003 | 0.0005 |
| *Brachybacterium* | 0.35443 | 0.56749 | 0.0000 | 0.0000 | 0.0000 | 0.0000 | 0.0000 | 0.0002 | 0.0001 |
| *Helicobacter* | 0.35706 | 0.56759 | 0.0007 | 0.0002 | 0.0003 | 0.0000 | 0.0001 | 0.0011 | 0.0017 |
| *Parabacteroides* | 0.35848 | 0.56759 | 0.0002 | 0.0006 | 0.0006 | 0.0000 | 0.0002 | 0.0027 | 0.0020 |
| *Lachnospiraceae FCS020 group* | 0.36517 | 0.57297 | 0.0002 | 0.0000 | 0.0000 | 0.0000 | 0.0000 | 0.0003 | 0.0000 |
| *Nocardioides* | 0.36959 | 0.57297 | 0.0001 | 0.0000 | 0.0000 | 0.0000 | 0.0000 | 0.0000 | 0.0003 |
| *Paracoccus* | 0.37182 | 0.57297 | 0.0003 | 0.0000 | 0.0000 | 0.0000 | 0.0000 | 0.0001 | 0.0000 |
| *ASF356* | 0.37184 | 0.57297 | 0.0001 | 0.0000 | 0.0002 | 0.0000 | 0.0000 | 0.0001 | 0.0005 |
| *A2* | 0.37269 | 0.57297 | 0.0004 | 0.0009 | 0.0002 | 0.0000 | 0.0000 | 0.0001 | 0.0006 |
| *Butyricicoccus* | 0.37394 | 0.57297 | 0.0000 | 0.0001 | 0.0000 | 0.0000 | 0.0000 | 0.0002 | 0.0005 |
| *Lachnospiraceae ND3007 group* | 0.38614 | 0.58850 | 0.0000 | 0.0000 | 0.0001 | 0.0000 | 0.0000 | 0.0004 | 0.0001 |
| *uncultured Bacteroidales bacterium* | 0.38858 | 0.58870 | 0.0006 | 0.0010 | 0.0003 | 0.0001 | 0.0000 | 0.0003 | 0.0014 |
| *Bosea* | 0.39046 | 0.58870 | 0.0001 | 0.0000 | 0.0000 | 0.0000 | 0.0000 | 0.0002 | 0.0000 |
| *Bacillus* | 0.39246 | 0.58870 | 0.0095 | 0.0002 | 0.0004 | 0.0002 | 0.0001 | 0.0004 | 0.0004 |
| *Anaerostipes* | 0.39744 | 0.59003 | 0.0000 | 0.0000 | 0.0000 | 0.0000 | 0.0000 | 0.0004 | 0.0002 |
| *Erysipelatoclostridium* | 0.40584 | 0.59003 | 0.0000 | 0.0000 | 0.0001 | 0.0000 | 0.0000 | 0.0001 | 0.0001 |
| *Pseudogracilibacillus* | 0.41410 | 0.59003 | 0.0000 | 0.0001 | 0.0000 | 0.0001 | 0.0000 | 0.0002 | 0.0001 |
| *Ileibacterium* | 0.41692 | 0.59003 | 0.0003 | 0.0001 | 0.0000 | 0.0000 | 0.0000 | 0.0001 | 0.0000 |
| *Ruminiclostridium 5* | 0.42521 | 0.59003 | 0.0004 | 0.0000 | 0.0002 | 0.0001 | 0.0000 | 0.0004 | 0.0009 |
| *Lachnospiraceae NK4A136 group* | 0.42870 | 0.59003 | 0.0035 | 0.0027 | 0.0013 | 0.0006 | 0.0002 | 0.0020 | 0.0043 |
| *Lachnospiraceae UCG-006* | 0.43554 | 0.59003 | 0.0001 | 0.0002 | 0.0000 | 0.0000 | 0.0000 | 0.0003 | 0.0003 |
| *Marinifilum* | 0.43669 | 0.59003 | 0.0005 | 0.0000 | 0.0000 | 0.0001 | 0.0000 | 0.0000 | 0.0000 |
| *[Eubacterium] eligens group* | 0.44901 | 0.59003 | 0.0003 | 0.0000 | 0.0001 | 0.0000 | 0.0000 | 0.0003 | 0.0004 |
| *Chryseobacterium* | 0.45816 | 0.59003 | 0.0001 | 0.0000 | 0.0000 | 0.0000 | 0.0000 | 0.0000 | 0.0001 |
| *[Eubacterium] brachy group* | 0.45816 | 0.59003 | 0.0000 | 0.0000 | 0.0000 | 0.0000 | 0.0000 | 0.0001 | 0.0000 |
| *Commensalibacter* | 0.45816 | 0.59003 | 0.0000 | 0.0000 | 0.0000 | 0.0000 | 0.0000 | 0.0000 | 0.0001 |
| *Rikenella* | 0.45826 | 0.59003 | 0.0002 | 0.0002 | 0.0000 | 0.0000 | 0.0000 | 0.0000 | 0.0004 |
| *Coprococcus 2* | 0.47153 | 0.59003 | 0.0001 | 0.0000 | 0.0001 | 0.0000 | 0.0000 | 0.0000 | 0.0000 |
| *Rothia* | 0.47464 | 0.59003 | 0.0000 | 0.0000 | 0.0000 | 0.0000 | 0.0001 | 0.0000 | 0.0002 |
| *[Eubacterium] fissicatena group* | 0.47745 | 0.59003 | 0.0000 | 0.0000 | 0.0000 | 0.0000 | 0.0000 | 0.0001 | 0.0000 |
| *[Eubacterium] ruminantium group* | 0.47745 | 0.59003 | 0.0000 | 0.0000 | 0.0000 | 0.0000 | 0.0000 | 0.0000 | 0.0001 |
| *Other* | 0.49713 | 0.59003 | 0.1188 | 0.0074 | 0.0052 | 0.0019 | 0.0025 | 0.0147 | 0.0558 |
| *Nitratireductor* | 0.50524 | 0.59003 | 0.0000 | 0.0000 | 0.0002 | 0.0001 | 0.0001 | 0.0000 | 0.0000 |
| *Klebsiella* | 0.50881 | 0.59003 | 0.0000 | 0.0000 | 0.0000 | 0.0001 | 0.0271 | 0.0000 | 0.0000 |
| *Rheinheimera* | 0.51095 | 0.59003 | 0.0006 | 0.0000 | 0.0000 | 0.0000 | 0.0000 | 0.0000 | 0.0000 |
| *Wolbachia* | 0.51095 | 0.59003 | 0.0000 | 0.0000 | 0.0000 | 0.0005 | 0.0000 | 0.0000 | 0.0000 |
| *Moraxella* | 0.51095 | 0.59003 | 0.0000 | 0.0004 | 0.0000 | 0.0000 | 0.0000 | 0.0000 | 0.0000 |
| *Rodentibacter* | 0.51095 | 0.59003 | 0.0000 | 0.0000 | 0.0000 | 0.0000 | 0.0003 | 0.0000 | 0.0000 |
| *Schlegelella* | 0.51095 | 0.59003 | 0.0002 | 0.0000 | 0.0000 | 0.0000 | 0.0000 | 0.0000 | 0.0000 |
| *uncultured prokaryote* | 0.51095 | 0.59003 | 0.0000 | 0.0001 | 0.0000 | 0.0000 | 0.0000 | 0.0000 | 0.0000 |
| *Hypnocyclicus* | 0.51095 | 0.59003 | 0.0000 | 0.0000 | 0.0000 | 0.0001 | 0.0000 | 0.0000 | 0.0000 |
| *Virgibacillus* | 0.51095 | 0.59003 | 0.0000 | 0.0001 | 0.0000 | 0.0000 | 0.0000 | 0.0000 | 0.0000 |
| *Arcicella* | 0.51095 | 0.59003 | 0.0001 | 0.0000 | 0.0000 | 0.0000 | 0.0000 | 0.0000 | 0.0000 |
| *Aquabacterium* | 0.51095 | 0.59003 | 0.0001 | 0.0000 | 0.0000 | 0.0000 | 0.0000 | 0.0000 | 0.0000 |
| *Porphyromonas* | 0.51095 | 0.59003 | 0.0000 | 0.0000 | 0.0001 | 0.0000 | 0.0000 | 0.0000 | 0.0000 |
| *Aestuariicella* | 0.51095 | 0.59003 | 0.0000 | 0.0001 | 0.0000 | 0.0000 | 0.0000 | 0.0000 | 0.0000 |
| *Photobacterium* | 0.51095 | 0.59003 | 0.0000 | 0.0000 | 0.0000 | 0.0001 | 0.0000 | 0.0000 | 0.0000 |
| *uncultured forest soil bacterium* | 0.51095 | 0.59003 | 0.0000 | 0.0001 | 0.0000 | 0.0000 | 0.0000 | 0.0000 | 0.0000 |
| *Myroides* | 0.51095 | 0.59003 | 0.0000 | 0.0001 | 0.0000 | 0.0000 | 0.0000 | 0.0000 | 0.0000 |
| *Flavobacterium* | 0.51095 | 0.59003 | 0.0000 | 0.0000 | 0.0001 | 0.0000 | 0.0000 | 0.0000 | 0.0000 |
| *Corynebacterium* | 0.51095 | 0.59003 | 0.0001 | 0.0000 | 0.0000 | 0.0000 | 0.0000 | 0.0000 | 0.0000 |
| *Lachnospiraceae NK3A20 group* | 0.51095 | 0.59003 | 0.0000 | 0.0000 | 0.0000 | 0.0000 | 0.0000 | 0.0000 | 0.0000 |
| *Bauldia* | 0.51095 | 0.59003 | 0.0000 | 0.0000 | 0.0000 | 0.0000 | 0.0000 | 0.0000 | 0.0000 |
| *Hyphomicrobium* | 0.51095 | 0.59003 | 0.0000 | 0.0000 | 0.0000 | 0.0000 | 0.0000 | 0.0000 | 0.0000 |
| *Pajaroellobacter* | 0.51095 | 0.59003 | 0.0000 | 0.0000 | 0.0000 | 0.0000 | 0.0000 | 0.0000 | 0.0000 |
| *Flectobacillus* | 0.51095 | 0.59003 | 0.0000 | 0.0000 | 0.0000 | 0.0000 | 0.0000 | 0.0000 | 0.0000 |
| *Permianibacter* | 0.51095 | 0.59003 | 0.0000 | 0.0000 | 0.0000 | 0.0000 | 0.0000 | 0.0000 | 0.0000 |
| *Crocinitomix* | 0.51095 | 0.59003 | 0.0000 | 0.0000 | 0.0000 | 0.0000 | 0.0000 | 0.0000 | 0.0000 |
| *Buchnera* | 0.51095 | 0.59003 | 0.0000 | 0.0000 | 0.0000 | 0.0000 | 0.0000 | 0.0000 | 0.0000 |
| *Nakamurella* | 0.51095 | 0.59003 | 0.0000 | 0.0000 | 0.0000 | 0.0000 | 0.0000 | 0.0000 | 0.0000 |
| *Mitsuokella* | 0.51095 | 0.59003 | 0.0000 | 0.0000 | 0.0000 | 0.0000 | 0.0000 | 0.0000 | 0.0000 |
| *Stenotrophomonas* | 0.51095 | 0.59003 | 0.0000 | 0.0000 | 0.0000 | 0.0000 | 0.0000 | 0.0000 | 0.0000 |
| *Marinobacter* | 0.51095 | 0.59003 | 0.0000 | 0.0000 | 0.0000 | 0.0000 | 0.0000 | 0.0000 | 0.0000 |
| *Kistimonas* | 0.51095 | 0.59003 | 0.0000 | 0.0000 | 0.0000 | 0.0000 | 0.0000 | 0.0000 | 0.0000 |
| *Symphothece PCC-7002* | 0.51095 | 0.59003 | 0.0000 | 0.0000 | 0.0000 | 0.0000 | 0.0000 | 0.0000 | 0.0000 |
| *Catonella* | 0.51095 | 0.59003 | 0.0000 | 0.0000 | 0.0000 | 0.0000 | 0.0000 | 0.0000 | 0.0000 |
| *Tissierella* | 0.51095 | 0.59003 | 0.0000 | 0.0000 | 0.0000 | 0.0000 | 0.0000 | 0.0000 | 0.0000 |
| *Rhodococcus* | 0.51095 | 0.59003 | 0.0000 | 0.0000 | 0.0000 | 0.0000 | 0.0000 | 0.0000 | 0.0000 |
| *Rhodovulum* | 0.51095 | 0.59003 | 0.0000 | 0.0000 | 0.0000 | 0.0000 | 0.0000 | 0.0000 | 0.0000 |
| *Veillonella* | 0.51095 | 0.59003 | 0.0000 | 0.0000 | 0.0000 | 0.0000 | 0.0000 | 0.0000 | 0.0000 |
| *Anaeroplasma* | 0.51136 | 0.59003 | 0.0008 | 0.0004 | 0.0000 | 0.0001 | 0.0000 | 0.0001 | 0.0002 |
| *GCA-900066225* | 0.51622 | 0.59324 | 0.0000 | 0.0000 | 0.0000 | 0.0000 | 0.0000 | 0.0001 | 0.0001 |
| *Mucispirillum* | 0.52323 | 0.59515 | 0.0008 | 0.0004 | 0.0002 | 0.0000 | 0.0001 | 0.0001 | 0.0000 |
| *Prevotellaceae UCG-001* | 0.52348 | 0.59515 | 0.0003 | 0.0004 | 0.0005 | 0.0002 | 0.0002 | 0.0005 | 0.0017 |
| *Streptococcus* | 0.52551 | 0.59515 | 0.0001 | 0.0001 | 0.0002 | 0.0001 | 0.0001 | 0.0005 | 0.0005 |
| *Streptomyces* | 0.52624 | 0.59515 | 0.0001 | 0.0000 | 0.0002 | 0.0000 | 0.0000 | 0.0002 | 0.0005 |
| *Delftia* | 0.53574 | 0.59968 | 0.0000 | 0.0000 | 0.0000 | 0.0000 | 0.0001 | 0.0000 | 0.0001 |
| *Finegoldia* | 0.53574 | 0.59968 | 0.0001 | 0.0000 | 0.0000 | 0.0000 | 0.0000 | 0.0000 | 0.0001 |
| *Prevotella 9* | 0.53656 | 0.59968 | 0.0047 | 0.0012 | 0.0063 | 0.0012 | 0.0000 | 0.0009 | 0.0032 |
| *Methylobacterium* | 0.54328 | 0.60483 | 0.0003 | 0.0000 | 0.0000 | 0.0000 | 0.0000 | 0.0001 | 0.0002 |
| *Ruminiclostridium 9* | 0.56046 | 0.62152 | 0.0001 | 0.0004 | 0.0003 | 0.0001 | 0.0000 | 0.0003 | 0.0004 |
| *Atopobium* | 0.56573 | 0.62371 | 0.0000 | 0.0000 | 0.0002 | 0.0000 | 0.0000 | 0.0000 | 0.0000 |
| *Ellin6055* | 0.56750 | 0.62371 | 0.0002 | 0.0000 | 0.0000 | 0.0000 | 0.0000 | 0.0001 | 0.0002 |
| *Mycobacterium* | 0.56900 | 0.62371 | 0.0002 | 0.0000 | 0.0000 | 0.0006 | 0.0000 | 0.0000 | 0.0000 |
| *Bilophila* | 0.57885 | 0.63208 | 0.0007 | 0.0004 | 0.0002 | 0.0000 | 0.0001 | 0.0003 | 0.0002 |
| *Parvibaculum* | 0.58129 | 0.63232 | 0.0000 | 0.0000 | 0.0001 | 0.0000 | 0.0000 | 0.0000 | 0.0000 |
| *Candidatus Stoquefichus* | 0.60571 | 0.65116 | 0.0001 | 0.0000 | 0.0000 | 0.0000 | 0.0000 | 0.0000 | 0.0000 |
| *Campylobacter* | 0.60571 | 0.65116 | 0.0000 | 0.0000 | 0.0000 | 0.0001 | 0.0000 | 0.0000 | 0.0000 |
| *Ruminiclostridium* | 0.60698 | 0.65116 | 0.0003 | 0.0002 | 0.0002 | 0.0000 | 0.0000 | 0.0001 | 0.0001 |
| *Ralstonia* | 0.60835 | 0.65116 | 0.0000 | 0.0002 | 0.0001 | 0.0000 | 0.0000 | 0.0000 | 0.0000 |
| *Fabibacter* | 0.61228 | 0.65116 | 0.0004 | 0.0003 | 0.0002 | 0.0002 | 0.0001 | 0.0000 | 0.0000 |
| *Acidibacter* | 0.61232 | 0.65116 | 0.0003 | 0.0000 | 0.0000 | 0.0000 | 0.0000 | 0.0001 | 0.0001 |
| *Xanthomonas* | 0.63051 | 0.66554 | 0.0000 | 0.0000 | 0.0000 | 0.0000 | 0.0000 | 0.0000 | 0.0000 |
| *Prevotella* | 0.63051 | 0.66554 | 0.0000 | 0.0000 | 0.0000 | 0.0000 | 0.0000 | 0.0000 | 0.0000 |
| *Paraprevotella* | 0.63713 | 0.67005 | 0.0003 | 0.0000 | 0.0002 | 0.0000 | 0.0000 | 0.0000 | 0.0001 |
| *Brevibacterium* | 0.64411 | 0.67489 | 0.0000 | 0.0002 | 0.0000 | 0.0000 | 0.0000 | 0.0001 | 0.0000 |
| *Treponema 2* | 0.65213 | 0.67665 | 0.0000 | 0.0000 | 0.0001 | 0.0000 | 0.0000 | 0.0001 | 0.0001 |
| *Sphingopyxis* | 0.65447 | 0.67665 | 0.0000 | 0.0001 | 0.0000 | 0.0000 | 0.0000 | 0.0001 | 0.0000 |
| *Dubosiella* | 0.65447 | 0.67665 | 0.0001 | 0.0000 | 0.0000 | 0.0000 | 0.0000 | 0.0000 | 0.0001 |
| *Haliangium* | 0.65528 | 0.67665 | 0.0002 | 0.0000 | 0.0001 | 0.0000 | 0.0000 | 0.0000 | 0.0001 |
| *Shinella* | 0.67054 | 0.68990 | 0.0001 | 0.0000 | 0.0000 | 0.0000 | 0.0000 | 0.0001 | 0.0001 |
| *Weissella* | 0.67997 | 0.69709 | 0.0000 | 0.0000 | 0.0002 | 0.0000 | 0.0000 | 0.0001 | 0.0000 |
| *Sutterella* | 0.70350 | 0.71863 | 0.0003 | 0.0000 | 0.0002 | 0.0000 | 0.0000 | 0.0001 | 0.0002 |
| *Azotobacter* | 0.72398 | 0.73691 | 0.0000 | 0.0000 | 0.0001 | 0.0001 | 0.0000 | 0.0000 | 0.0000 |
| *Enterobacter* | 0.74814 | 0.75879 | 0.0003 | 0.1119 | 0.0007 | 0.1309 | 0.1096 | 0.0001 | 0.0000 |
| *Hyphobacterium* | 0.76904 | 0.77722 | 0.0001 | 0.0001 | 0.0002 | 0.0001 | 0.0001 | 0.0000 | 0.0000 |
| *Rikenellaceae RC9 gut group* | 0.77234 | 0.77780 | 0.0004 | 0.0004 | 0.0007 | 0.0003 | 0.0001 | 0.0002 | 0.0005 |
| *Enterorhabdus* | 0.79478 | 0.79758 | 0.0000 | 0.0000 | 0.0000 | 0.0000 | 0.0000 | 0.0000 | 0.0001 |
| *Lachnospiraceae UCG-001* | 0.83450 | 0.83450 | 0.0001 | 0.0002 | 0.0002 | 0.0001 | 0.0000 | 0.0002 | 0.0002 |
