# Supplementary material for: Dynamic of Composition and Diversity of Gut Microbiota in Triatoma rubrofasciata in Different Developmental Stages and Environmental Conditions
Source: Front Cell Infect Microbiol. 2020 Nov 2;10:587708. doi: 10.3389/fcimb.2020.587708 (PMC7667259; doi:10.3389/fcimb.2020.587708)
Supplement: Supplementary Table 1 — OTU tables and taxonomic classifications of the 16S rRNA gene. [file DataSheet_1.zip › Supplementary Table S5.DOCX]

| **Phylum** | ***p*-value** | **FDR** | **Lab** | **Wild** |
| --- | --- | --- | --- | --- |
| Proteobacteria | 0.00886 | 0.28353 | 0.2027 | 0.7975 |
| Firmicutes | 0.08576 | 0.60538 | 0.5881 | 0.1730 |
| Epsilonbacteraeota | 0.14479 | 0.60538 | 0.0008 | 0.0000 |
| Deferribacteres | 0.19272 | 0.60538 | 0.0002 | 0.0000 |
| Actinobacteria | 0.19887 | 0.60538 | 0.0287 | 0.0018 |
| Nitrospirae | 0.25858 | 0.60538 | 0.0002 | 0.0000 |
| Acidobacteria | 0.26266 | 0.60538 | 0.0011 | 0.0000 |
| Gemmatimonadetes | 0.27719 | 0.60538 | 0.0004 | 0.0000 |
| Spirochaetes | 0.30988 | 0.60538 | 0.0001 | 0.0000 |
| Nanoarchaeaeota | 0.35819 | 0.60538 | 0.0001 | 0.0000 |
| Thaumarchaeota | 0.36129 | 0.60538 | 0.0128 | 0.0000 |
| Cyanobacteria | 0.36367 | 0.60538 | 0.0002 | 0.0000 |
| Crenarchaeota | 0.36833 | 0.60538 | 0.0783 | 0.0000 |
| Euryarchaeota | 0.37067 | 0.60538 | 0.0458 | 0.0000 |
| Planctomycetes | 0.37278 | 0.60538 | 0.0000 | 0.0000 |
| Diapherotrites | 0.37396 | 0.60538 | 0.0009 | 0.0000 |
| Asgardaeota | 0.40789 | 0.60538 | 0.0002 | 0.0000 |
| Altiarchaeota | 0.41735 | 0.60538 | 0.0019 | 0.0000 |
| Elusimicrobia | 0.43475 | 0.60538 | 0.0000 | 0.0000 |
| Chloroflexi | 0.43475 | 0.60538 | 0.0000 | 0.0000 |
| Other | 0.44208 | 0.60538 | 0.0047 | 0.0004 |
| WS1 | 0.45685 | 0.60538 | 0.0000 | 0.0000 |
| Tenericutes | 0.49162 | 0.60538 | 0.0001 | 0.0000 |
| Lentisphaerae | 0.54863 | 0.60538 | 0.0000 | 0.0000 |
| Fibrobacteres | 0.54863 | 0.60538 | 0.0000 | 0.0000 |
| Omnitrophicaeota | 0.54863 | 0.60538 | 0.0000 | 0.0000 |
| GAL15 | 0.54863 | 0.60538 | 0.0000 | 0.0000 |
| Deinococcus-Thermus | 0.54863 | 0.60538 | 0.0000 | 0.0000 |
| Verrucomicrobia | 0.54863 | 0.60538 | 0.0000 | 0.0000 |
| Bacteroidetes | 0.78837 | 0.84093 | 0.0322 | 0.0270 |
| Patescibacteria | 0.90332 | 0.93246 | 0.0001 | 0.0001 |
| Fusobacteria | 0.96546 | 0.96546 | 0.0002 | 0.0002 |
