# Supplementary material for: Dynamic of Composition and Diversity of Gut Microbiota in Triatoma rubrofasciata in Different Developmental Stages and Environmental Conditions
Source: Front Cell Infect Microbiol. 2020 Nov 2;10:587708. doi: 10.3389/fcimb.2020.587708 (PMC7667259; doi:10.3389/fcimb.2020.587708)
Supplement: Supplementary Table 1 — OTU tables and taxonomic classifications of the 16S rRNA gene. [file DataSheet_1.zip › Supplementary Table S6.DOCX]

| **Genus** | ***p*-value** | **FDR** | **Lab** | **Wild** |
| --- | --- | --- | --- | --- |
| *Peptoniphilus* | 0.00076 | 0.16389 | 0.0000 | 0.0012 |
| *Other* | 0.00091 | 0.16389 | 0.0451 | 0.6285 |
| *Staphylococcus* | 0.02760 | 0.55795 | 0.5498 | 0.0023 |
| *Enterococcus* | 0.04122 | 0.55795 | 0.0007 | 0.0058 |
| *uncultured organism* | 0.05121 | 0.55795 | 0.0003 | 0.0000 |
| *Fusicatenibacter* | 0.06545 | 0.55795 | 0.0002 | 0.0000 |
| *[Ruminococcus] torques group* | 0.06946 | 0.55795 | 0.0009 | 0.0000 |
| *uncultured Bacteroidales bacterium* | 0.07941 | 0.55795 | 0.0005 | 0.0000 |
| *[Eubacterium] eligens group* | 0.08214 | 0.55795 | 0.0001 | 0.0000 |
| *Ruminococcus 2* | 0.08686 | 0.55795 | 0.0006 | 0.0000 |
| *Odoribacter* | 0.08913 | 0.55795 | 0.0006 | 0.0000 |
| *Faecalibacterium* | 0.09397 | 0.55795 | 0.0013 | 0.0000 |
| *Ruminiclostridium 9* | 0.10420 | 0.55795 | 0.0001 | 0.0000 |
| *Ruminiclostridium 5* | 0.11024 | 0.55795 | 0.0002 | 0.0000 |
| *Pantoea* | 0.11278 | 0.55795 | 0.0000 | 0.2260 |
| *Bacillus* | 0.11437 | 0.55795 | 0.0002 | 0.1219 |
| *Intestinimonas* | 0.11504 | 0.55795 | 0.0001 | 0.0000 |
| *Exiguobacterium* | 0.11676 | 0.55795 | 0.0000 | 0.0000 |
| *Alistipes* | 0.11851 | 0.55795 | 0.0023 | 0.0002 |
| *Marmoricola* | 0.11980 | 0.55795 | 0.0001 | 0.0000 |
| *Bacteroides* | 0.12323 | 0.55795 | 0.0081 | 0.0004 |
| *Dorea* | 0.13497 | 0.55795 | 0.0004 | 0.0000 |
| *Alloprevotella* | 0.13814 | 0.55795 | 0.0022 | 0.0000 |
| *Helicobacter* | 0.14090 | 0.55795 | 0.0008 | 0.0000 |
| *uncultured* | 0.14553 | 0.55795 | 0.0019 | 0.0000 |
| *Subdoligranulum* | 0.14778 | 0.55795 | 0.0008 | 0.0000 |
| *Ruminococcus 1* | 0.14935 | 0.55795 | 0.0006 | 0.0000 |
| *Lachnospiraceae NK4A136 group* | 0.15416 | 0.55795 | 0.0023 | 0.0001 |
| *Barnesiella* | 0.15551 | 0.55795 | 0.0004 | 0.0000 |
| *uncultured forest soil bacterium* | 0.15645 | 0.55795 | 0.0000 | 0.0000 |
| *Prevotellaceae UCG-001* | 0.15726 | 0.55795 | 0.0005 | 0.0000 |
| *Rikenellaceae RC9 gut group* | 0.15927 | 0.55795 | 0.0003 | 0.0000 |
| *Lactobacillus* | 0.16105 | 0.55795 | 0.0031 | 0.0000 |
| *Roseburia* | 0.16563 | 0.55795 | 0.0010 | 0.0001 |
| *Gaiella* | 0.16802 | 0.55795 | 0.0002 | 0.0000 |
| *Atopostipes* | 0.16802 | 0.55795 | 0.0001 | 0.0000 |
| *Ruminococcaceae UCG-003* | 0.16893 | 0.55795 | 0.0001 | 0.0000 |
| *Collinsella* | 0.17256 | 0.55795 | 0.0007 | 0.0000 |
| *Marvinbryantia* | 0.17368 | 0.55795 | 0.0001 | 0.0000 |
| *Family XIII AD3011 group* | 0.17540 | 0.55795 | 0.0001 | 0.0000 |
| *Senegalimassilia* | 0.17540 | 0.55795 | 0.0001 | 0.0000 |
| *Parasutterella* | 0.18011 | 0.55795 | 0.0002 | 0.0000 |
| *Pseudogracilibacillus* | 0.18692 | 0.55795 | 0.0002 | 0.0000 |
| *Mucispirillum* | 0.19272 | 0.55795 | 0.0002 | 0.0000 |
| *Ellin6067* | 0.19272 | 0.55795 | 0.0002 | 0.0000 |
| *uncultured Porphyromonadaceae bacterium* | 0.19272 | 0.55795 | 0.0000 | 0.0000 |
| *Ruminococcaceae UCG-002* | 0.19397 | 0.55795 | 0.0013 | 0.0000 |
| *Ruminococcaceae UCG-005* | 0.19445 | 0.55795 | 0.0012 | 0.0000 |
| *Glycomyces* | 0.19723 | 0.55795 | 0.0001 | 0.0000 |
| *Erysipelotrichaceae UCG-003* | 0.20298 | 0.55795 | 0.0001 | 0.0000 |
| *Bradyrhizobium* | 0.20666 | 0.55795 | 0.0002 | 0.0000 |
| *Acinetobacter* | 0.21492 | 0.55795 | 0.0007 | 0.0028 |
| *[Eubacterium] ventriosum group* | 0.21495 | 0.55795 | 0.0001 | 0.0000 |
| *[Eubacterium] coprostanoligenes group* | 0.21618 | 0.55795 | 0.0026 | 0.0000 |
| *Massilia* | 0.21876 | 0.55795 | 0.0003 | 0.0000 |
| *Haliangium* | 0.22145 | 0.55795 | 0.0001 | 0.0000 |
| *Escherichia-Shigella* | 0.22496 | 0.55795 | 0.0019 | 0.0000 |
| *uncultured bacterium* | 0.22761 | 0.55795 | 0.0180 | 0.0001 |
| *Prevotella 9* | 0.23191 | 0.55795 | 0.0013 | 0.0000 |
| *Sphingomonas* | 0.24650 | 0.55795 | 0.0004 | 0.0000 |
| *Bosea* | 0.24877 | 0.55795 | 0.0000 | 0.0000 |
| *Paracoccus* | 0.24877 | 0.55795 | 0.0000 | 0.0000 |
| *Faecalitalea* | 0.25125 | 0.55795 | 0.0001 | 0.0000 |
| *Lysobacter* | 0.25508 | 0.55795 | 0.0001 | 0.0000 |
| *Phenylobacterium* | 0.25747 | 0.55795 | 0.0002 | 0.0000 |
| *Flavonifractor* | 0.25858 | 0.55795 | 0.0001 | 0.0000 |
| *Lachnospiraceae UCG-001* | 0.26035 | 0.55795 | 0.0002 | 0.0000 |
| *Prevotella 1* | 0.26530 | 0.55795 | 0.0001 | 0.0000 |
| *Coprococcus 3* | 0.26912 | 0.55795 | 0.0001 | 0.0000 |
| *Prevotellaceae Ga6A1 group* | 0.27069 | 0.55795 | 0.0001 | 0.0000 |
| *Treponema 2* | 0.27147 | 0.55795 | 0.0001 | 0.0000 |
| *Oceanobacillus* | 0.27321 | 0.55795 | 0.0001 | 0.0000 |
| *Sphingobium* | 0.27321 | 0.55795 | 0.0000 | 0.0000 |
| *Butyricimonas* | 0.27466 | 0.55795 | 0.0003 | 0.0000 |
| *Lachnoclostridium* | 0.27772 | 0.55795 | 0.0005 | 0.0000 |
| *Parabacteroides* | 0.28000 | 0.55795 | 0.0008 | 0.0000 |
| *Desulfovibrio* | 0.28458 | 0.55795 | 0.0007 | 0.0000 |
| *Nocardioides* | 0.28603 | 0.55795 | 0.0000 | 0.0000 |
| *Ruminococcaceae UCG-010* | 0.28603 | 0.55795 | 0.0000 | 0.0000 |
| *Candidatus Solibacter* | 0.28834 | 0.55795 | 0.0001 | 0.0000 |
| *Pseudarthrobacter* | 0.28926 | 0.55795 | 0.0002 | 0.0000 |
| *Nitrospira* | 0.28976 | 0.55795 | 0.0002 | 0.0000 |
| *Dialister* | 0.29350 | 0.55795 | 0.0002 | 0.0000 |
| *endosymbionts8* | 0.29428 | 0.55795 | 0.0002 | 0.0000 |
| *Bryobacter* | 0.29428 | 0.55795 | 0.0002 | 0.0000 |
| *Anaerotruncus* | 0.29646 | 0.55795 | 0.0001 | 0.0000 |
| *Dubosiella* | 0.29646 | 0.55795 | 0.0001 | 0.0000 |
| *Oscillibacter* | 0.29737 | 0.55795 | 0.0001 | 0.0000 |
| *[Eubacterium] xylanophilum group* | 0.30582 | 0.55795 | 0.0003 | 0.0000 |
| *Eisenbergiella* | 0.30948 | 0.55795 | 0.0004 | 0.0000 |
| *Turicibacter* | 0.31113 | 0.55795 | 0.0006 | 0.0000 |
| *Christensenellaceae R-7 group* | 0.31296 | 0.55795 | 0.0005 | 0.0000 |
| *Allobaculum* | 0.31985 | 0.55795 | 0.0003 | 0.0000 |
| *Muribaculum* | 0.32103 | 0.55795 | 0.0001 | 0.0000 |
| *Hungatella* | 0.32112 | 0.55795 | 0.0002 | 0.0000 |
| *MND1* | 0.32800 | 0.55795 | 0.0002 | 0.0000 |
| *Blautia* | 0.32919 | 0.55795 | 0.0010 | 0.0001 |
| *Rikenella* | 0.32954 | 0.55795 | 0.0001 | 0.0000 |
| *Ruminococcaceae UCG-004* | 0.32965 | 0.55795 | 0.0002 | 0.0000 |
| *Streptococcus* | 0.32965 | 0.55795 | 0.0001 | 0.0000 |
| *Coprobacillus* | 0.33099 | 0.55795 | 0.0002 | 0.0000 |
| *Solirubrobacter* | 0.33339 | 0.55795 | 0.0001 | 0.0000 |
| *Hydrogenophaga* | 0.33511 | 0.55795 | 0.0000 | 0.0000 |
| *Clostridium sensu stricto 1* | 0.34693 | 0.55795 | 0.0012 | 0.0000 |
| *Candidatus Methanomethylicus* | 0.35333 | 0.55795 | 0.0002 | 0.0000 |
| *[Ruminococcus] gnavus group* | 0.35348 | 0.55795 | 0.0003 | 0.0000 |
| *Lachnospiraceae UCG-010* | 0.35395 | 0.55795 | 0.0001 | 0.0000 |
| *Ohtaekwangia* | 0.35395 | 0.55795 | 0.0001 | 0.0000 |
| *Ruminococcaceae NK4A214 group* | 0.35422 | 0.55795 | 0.0008 | 0.0000 |
| *Rice Cluster I* | 0.35527 | 0.55795 | 0.0018 | 0.0000 |
| *Candidatus Nitrososphaera* | 0.35949 | 0.55795 | 0.0017 | 0.0000 |
| *Aeromonas* | 0.36048 | 0.55795 | 0.0001 | 0.0000 |
| *Methanocella* | 0.36347 | 0.55795 | 0.0017 | 0.0000 |
| *Methanosarcina* | 0.36396 | 0.55795 | 0.0017 | 0.0000 |
| *Prevotella 7* | 0.36550 | 0.55795 | 0.0001 | 0.0000 |
| *Methanomassiliicoccus* | 0.36772 | 0.55795 | 0.0029 | 0.0000 |
| *uncultured methanogenic archaeon* | 0.36942 | 0.55795 | 0.0445 | 0.0000 |
| *Candidatus Nitrosotalea* | 0.36967 | 0.55795 | 0.0023 | 0.0000 |
| *Methanosaeta* | 0.37120 | 0.55795 | 0.0021 | 0.0000 |
| *uncultured archaeon* | 0.37166 | 0.55795 | 0.0054 | 0.0000 |
| *Gemmatimonas* | 0.37278 | 0.55795 | 0.0001 | 0.0000 |
| *Allorhizobium-Neorhizobium-Pararhizobium-Rhizobium* | 0.37278 | 0.55795 | 0.0001 | 0.0000 |
| *Hymenobacter* | 0.37278 | 0.55795 | 0.0001 | 0.0000 |
| *Erysipelatoclostridium* | 0.37278 | 0.55795 | 0.0001 | 0.0000 |
| *Clostridium sensu stricto 10* | 0.37278 | 0.55795 | 0.0001 | 0.0000 |
| *Methylobacterium* | 0.37278 | 0.55795 | 0.0001 | 0.0000 |
| *Acidovorax* | 0.37278 | 0.55795 | 0.0001 | 0.0000 |
| *Azoarcus* | 0.37278 | 0.55795 | 0.0000 | 0.0000 |
| *Sutterella* | 0.37278 | 0.55795 | 0.0000 | 0.0000 |
| *Blvii28 wastewater-sludge group* | 0.37278 | 0.55795 | 0.0000 | 0.0000 |
| *Campylobacter* | 0.37278 | 0.55795 | 0.0000 | 0.0000 |
| *[Eubacterium] ruminantium group* | 0.37278 | 0.55795 | 0.0000 | 0.0000 |
| *Aquabacterium* | 0.37278 | 0.55795 | 0.0000 | 0.0000 |
| *Aetherobacter* | 0.37278 | 0.55795 | 0.0000 | 0.0000 |
| *Agromyces* | 0.37278 | 0.55795 | 0.0000 | 0.0000 |
| *Steroidobacter* | 0.37278 | 0.55795 | 0.0000 | 0.0000 |
| *Candidatus Koribacter* | 0.37278 | 0.55795 | 0.0000 | 0.0000 |
| *Mucilaginibacter* | 0.37278 | 0.55795 | 0.0000 | 0.0000 |
| *Megamonas* | 0.37278 | 0.55795 | 0.0000 | 0.0000 |
| *Nitrosospira* | 0.37278 | 0.55795 | 0.0000 | 0.0000 |
| *Nonomuraea* | 0.37278 | 0.55795 | 0.0000 | 0.0000 |
| *Neisseria* | 0.37278 | 0.55795 | 0.0000 | 0.0000 |
| *Pseudokineococcus* | 0.37278 | 0.55795 | 0.0000 | 0.0000 |
| *Rhodoplanes* | 0.37278 | 0.55795 | 0.0000 | 0.0000 |
| *Nakamurella* | 0.37278 | 0.55795 | 0.0000 | 0.0000 |
| *Phascolarctobacterium* | 0.37278 | 0.55795 | 0.0000 | 0.0000 |
| *Hyphomicrobium* | 0.37278 | 0.55795 | 0.0000 | 0.0000 |
| *Clostridium sensu stricto 11* | 0.37278 | 0.55795 | 0.0000 | 0.0000 |
| *Candidatus Methanoperedens* | 0.37306 | 0.55795 | 0.0026 | 0.0000 |
| *uncultured crenarchaeote* | 0.37406 | 0.55795 | 0.0001 | 0.0000 |
| *Bifidobacterium* | 0.37515 | 0.55795 | 0.0013 | 0.0000 |
| *Methanobacterium* | 0.37528 | 0.55795 | 0.0300 | 0.0000 |
| *[Clostridium] innocuum group* | 0.37690 | 0.55795 | 0.0002 | 0.0000 |
| *Lachnospiraceae UCG-006* | 0.37725 | 0.55795 | 0.0003 | 0.0000 |
| *Rhizobacter* | 0.37860 | 0.55795 | 0.0001 | 0.0000 |
| *OM27 clade* | 0.38159 | 0.55795 | 0.0002 | 0.0000 |
| *Chryseobacterium* | 0.38404 | 0.55795 | 0.0000 | 0.0001 |
| *Methanobrevibacter* | 0.38404 | 0.55795 | 0.0002 | 0.0000 |
| *Candidatus Nitrosotenuis* | 0.38404 | 0.55795 | 0.0001 | 0.0000 |
| *Dongia* | 0.38404 | 0.55795 | 0.0001 | 0.0000 |
| *uncultured Bathyarchaeota archaeon* | 0.38404 | 0.55795 | 0.0001 | 0.0000 |
| *Methanoregula* | 0.38404 | 0.55795 | 0.0000 | 0.0000 |
| *Flavisolibacter* | 0.38404 | 0.55795 | 0.0000 | 0.0000 |
| *Conexibacter* | 0.38476 | 0.55795 | 0.0003 | 0.0000 |
| *[Eubacterium] hallii group* | 0.38758 | 0.55795 | 0.0003 | 0.0000 |
| *Adlercreutzia* | 0.38850 | 0.55795 | 0.0007 | 0.0000 |
| *Perlucidibaca* | 0.39325 | 0.55795 | 0.0001 | 0.0000 |
| *Ruminococcaceae UCG-014* | 0.39526 | 0.55795 | 0.0024 | 0.0000 |
| *Pseudomonas* | 0.39666 | 0.55795 | 0.0002 | 0.0000 |
| *Romboutsia* | 0.39912 | 0.55795 | 0.0017 | 0.0000 |
| *Rhodanobacter* | 0.40038 | 0.55795 | 0.0001 | 0.0000 |
| *Plesiomonas* | 0.40267 | 0.55795 | 0.0001 | 0.0000 |
| *AKIW659* | 0.40267 | 0.55795 | 0.0000 | 0.0000 |
| *OLB12* | 0.40267 | 0.55795 | 0.0000 | 0.0000 |
| *Sneathia* | 0.40267 | 0.55795 | 0.0000 | 0.0000 |
| *Gemmatirosa* | 0.40267 | 0.55795 | 0.0000 | 0.0000 |
| *Caulobacter* | 0.40267 | 0.55795 | 0.0000 | 0.0000 |
| *Iamia* | 0.40267 | 0.55795 | 0.0000 | 0.0000 |
| *Haemophilus* | 0.40267 | 0.55795 | 0.0000 | 0.0000 |
| *Reyranella* | 0.40267 | 0.55795 | 0.0000 | 0.0000 |
| *Halomonas* | 0.40267 | 0.55795 | 0.0000 | 0.0000 |
| *Pygmaiobacter* | 0.40267 | 0.55795 | 0.0000 | 0.0000 |
| *Anaerovorax* | 0.40267 | 0.55795 | 0.0000 | 0.0000 |
| *Terrimonas* | 0.40267 | 0.55795 | 0.0000 | 0.0000 |
| *Methyloversatilis* | 0.40267 | 0.55795 | 0.0001 | 0.0000 |
| *archaeon GW2011 AR5* | 0.40267 | 0.55795 | 0.0001 | 0.0000 |
| *Parviterribacter* | 0.40681 | 0.55795 | 0.0015 | 0.0000 |
| *Enhydrobacter* | 0.41472 | 0.55795 | 0.0002 | 0.0000 |
| *uncultured haloarchaeon* | 0.41632 | 0.55795 | 0.0004 | 0.0000 |
| *Algoriphagus* | 0.42007 | 0.55795 | 0.0001 | 0.0000 |
| *Geobacter* | 0.42007 | 0.55795 | 0.0001 | 0.0000 |
| *Acidibacter* | 0.42095 | 0.55795 | 0.0001 | 0.0000 |
| *Noviherbaspirillum* | 0.42095 | 0.55795 | 0.0001 | 0.0000 |
| *Corynebacterium 1* | 0.42305 | 0.55795 | 0.0174 | 0.0004 |
| *Nocardiopsis* | 0.42711 | 0.55795 | 0.0002 | 0.0000 |
| *Ruminiclostridium 6* | 0.42801 | 0.55795 | 0.0002 | 0.0000 |
| *Shinella* | 0.43475 | 0.55795 | 0.0001 | 0.0000 |
| *Holdemanella* | 0.43475 | 0.55795 | 0.0001 | 0.0000 |
| *Anaeromyxobacter* | 0.43475 | 0.55795 | 0.0000 | 0.0000 |
| *Butyricicoccus* | 0.43475 | 0.55795 | 0.0000 | 0.0000 |
| *Clade Ib* | 0.43475 | 0.55795 | 0.0000 | 0.0000 |
| *Gordonibacter* | 0.43475 | 0.55795 | 0.0000 | 0.0000 |
| *uncultured Thermoplasmata archaeon* | 0.43475 | 0.55795 | 0.0000 | 0.0000 |
| *Motilibacter* | 0.43475 | 0.55795 | 0.0000 | 0.0000 |
| *Luedemannella* | 0.43475 | 0.55795 | 0.0000 | 0.0000 |
| *Acetobacter* | 0.43475 | 0.55795 | 0.0000 | 0.0000 |
| *Adhaeribacter* | 0.43475 | 0.55795 | 0.0000 | 0.0000 |
| *Streptomyces* | 0.44902 | 0.55795 | 0.0001 | 0.0000 |
| *UBA1819* | 0.44911 | 0.55795 | 0.0001 | 0.0000 |
| *Prevotella 2* | 0.44911 | 0.55795 | 0.0001 | 0.0000 |
| *Weissella* | 0.45685 | 0.55795 | 0.0001 | 0.0000 |
| *uncultured proteobacterium* | 0.45685 | 0.55795 | 0.0000 | 0.0000 |
| *Subgroup 10* | 0.45685 | 0.55795 | 0.0000 | 0.0000 |
| *Cellvibrio* | 0.45685 | 0.55795 | 0.0000 | 0.0000 |
| *Dechloromonas* | 0.45685 | 0.55795 | 0.0000 | 0.0000 |
| *Lachnospiraceae FCS020 group* | 0.45685 | 0.55795 | 0.0000 | 0.0000 |
| *Actinoplanes* | 0.45685 | 0.55795 | 0.0000 | 0.0000 |
| *Paludibaculum* | 0.45685 | 0.55795 | 0.0000 | 0.0000 |
| *Actinomadura* | 0.45685 | 0.55795 | 0.0000 | 0.0000 |
| *Anaeroplasma* | 0.45685 | 0.55795 | 0.0000 | 0.0000 |
| *Thauera* | 0.45685 | 0.55795 | 0.0000 | 0.0000 |
| *Clostridium sensu stricto 12* | 0.46007 | 0.55795 | 0.0002 | 0.0000 |
| *Jeotgalicoccus* | 0.46792 | 0.55795 | 0.0004 | 0.0000 |
| *uncultured euryarchaeote* | 0.47218 | 0.55795 | 0.0001 | 0.0000 |
| *A2* | 0.47218 | 0.55795 | 0.0001 | 0.0000 |
| *GCA-900066225* | 0.47218 | 0.55795 | 0.0001 | 0.0000 |
| *Rhodobacter* | 0.48327 | 0.55795 | 0.0001 | 0.0000 |
| *Enterorhabdus* | 0.48471 | 0.55795 | 0.0001 | 0.0000 |
| *Ambiguous taxa* | 0.48894 | 0.55795 | 0.1045 | 0.0085 |
| *Prevotellaceae NK3B31 group* | 0.48955 | 0.55795 | 0.0010 | 0.0000 |
| *Corynebacterium* | 0.49645 | 0.55795 | 0.0002 | 0.0000 |
| *Thermobifida* | 0.49810 | 0.55795 | 0.0001 | 0.0000 |
| *Paraprevotella* | 0.50711 | 0.55795 | 0.0000 | 0.0000 |
| *Chryseolinea* | 0.50750 | 0.55795 | 0.0001 | 0.0000 |
| *Enteractinococcus* | 0.51379 | 0.55795 | 0.0009 | 0.0000 |
| *Brachybacterium* | 0.52060 | 0.55795 | 0.0001 | 0.0000 |
| *Ruminiclostridium* | 0.52617 | 0.55795 | 0.0002 | 0.0000 |
| *Brevundimonas* | 0.54234 | 0.55795 | 0.0006 | 0.0000 |
| *Prevotellaceae UCG-003* | 0.54299 | 0.55795 | 0.0007 | 0.0000 |
| *Serratia* | 0.54795 | 0.55795 | 0.0895 | 0.0000 |
| *Hoyosella* | 0.54863 | 0.55795 | 0.0007 | 0.0000 |
| *Ruminococcaceae UCG-013* | 0.54863 | 0.55795 | 0.0002 | 0.0000 |
| *Vibrio* | 0.54863 | 0.55795 | 0.0001 | 0.0000 |
| *Flavobacterium* | 0.54863 | 0.55795 | 0.0001 | 0.0000 |
| *Pseudorhodobacter* | 0.54863 | 0.55795 | 0.0001 | 0.0000 |
| *Candidatus Saccharimonas* | 0.54863 | 0.55795 | 0.0001 | 0.0000 |
| *Saccharomonospora* | 0.54863 | 0.55795 | 0.0001 | 0.0000 |
| *Ferruginibacter* | 0.54863 | 0.55795 | 0.0001 | 0.0000 |
| *Pedobacter* | 0.54863 | 0.55795 | 0.0001 | 0.0000 |
| *Coprococcus 1* | 0.54863 | 0.55795 | 0.0000 | 0.0000 |
| *Tyzzerella 3* | 0.54863 | 0.55795 | 0.0000 | 0.0000 |
| *Dietzia* | 0.54863 | 0.55795 | 0.0000 | 0.0000 |
| *Rickettsia* | 0.54863 | 0.55795 | 0.0000 | 0.0000 |
| *Acetatifactor* | 0.54863 | 0.55795 | 0.0000 | 0.0000 |
| *Chthonobacter* | 0.54863 | 0.55795 | 0.0000 | 0.0000 |
| *Skermanella* | 0.54863 | 0.55795 | 0.0000 | 0.0000 |
| *uncultured Acidimicrobiales bacterium* | 0.54863 | 0.55795 | 0.0000 | 0.0000 |
| *Candidatus Alysiosphaera* | 0.54863 | 0.55795 | 0.0000 | 0.0000 |
| *Lewinella* | 0.54863 | 0.55795 | 0.0000 | 0.0000 |
| *Crossiella* | 0.54863 | 0.55795 | 0.0000 | 0.0000 |
| *Chlorobium* | 0.54863 | 0.55795 | 0.0000 | 0.0000 |
| *Rubellimicrobium* | 0.54863 | 0.55795 | 0.0000 | 0.0000 |
| *Tannerella* | 0.54863 | 0.55795 | 0.0000 | 0.0000 |
| *Hirschia* | 0.54863 | 0.55795 | 0.0000 | 0.0000 |
| *Pedomicrobium* | 0.54863 | 0.55795 | 0.0000 | 0.0000 |
| *[Eubacterium] yurii group* | 0.54863 | 0.55795 | 0.0000 | 0.0000 |
| *ASF356* | 0.54863 | 0.55795 | 0.0000 | 0.0000 |
| *Actinokineospora* | 0.54863 | 0.55795 | 0.0000 | 0.0000 |
| *Nordella* | 0.54863 | 0.55795 | 0.0000 | 0.0000 |
| *Polycyclovorans* | 0.54863 | 0.55795 | 0.0000 | 0.0000 |
| *Marinobacter* | 0.54863 | 0.55795 | 0.0000 | 0.0000 |
| *uncultured Acidobacteriales bacterium* | 0.54863 | 0.55795 | 0.0000 | 0.0000 |
| *mouse gut metagenome* | 0.54863 | 0.55795 | 0.0000 | 0.0000 |
| *Oscillospira* | 0.54863 | 0.55795 | 0.0000 | 0.0000 |
| *Bilophila* | 0.54863 | 0.55795 | 0.0000 | 0.0000 |
| *Olsenella* | 0.54863 | 0.55795 | 0.0000 | 0.0000 |
| *possible genus 04* | 0.54863 | 0.55795 | 0.0000 | 0.0000 |
| *Luteimonas* | 0.54863 | 0.55795 | 0.0000 | 0.0000 |
| *Fodinicola* | 0.54863 | 0.55795 | 0.0000 | 0.0000 |
| *Fluviicola* | 0.54863 | 0.55795 | 0.0000 | 0.0000 |
| *metagenome* | 0.54863 | 0.55795 | 0.0000 | 0.0000 |
| *Ureaplasma* | 0.54863 | 0.55795 | 0.0000 | 0.0000 |
| *Ureibacillus* | 0.54863 | 0.55795 | 0.0000 | 0.0000 |
| *Marinifilum* | 0.54863 | 0.55795 | 0.0000 | 0.0000 |
| *Arenimonas* | 0.54863 | 0.55795 | 0.0000 | 0.0000 |
| *hgcI clade* | 0.54863 | 0.55795 | 0.0000 | 0.0000 |
| *[Agitococcus] lubricus group* | 0.54863 | 0.55795 | 0.0000 | 0.0000 |
| *Granulicella* | 0.54863 | 0.55795 | 0.0000 | 0.0000 |
| *[Eubacterium] brachy group* | 0.54863 | 0.55795 | 0.0000 | 0.0000 |
| *Prevotella* | 0.54863 | 0.55795 | 0.0000 | 0.0000 |
| *Planifilum* | 0.54863 | 0.55795 | 0.0000 | 0.0000 |
| *Lactococcus* | 0.54863 | 0.55795 | 0.0000 | 0.0000 |
| *Tardiphaga* | 0.54863 | 0.55795 | 0.0000 | 0.0000 |
| *Variovorax* | 0.54863 | 0.55795 | 0.0000 | 0.0000 |
| *Devosia* | 0.54863 | 0.55795 | 0.0000 | 0.0000 |
| *Dokdonella* | 0.54863 | 0.55795 | 0.0000 | 0.0000 |
| *Clostridium sensu stricto 13* | 0.54863 | 0.55795 | 0.0000 | 0.0000 |
| *Nannocystis* | 0.54863 | 0.55795 | 0.0000 | 0.0000 |
| *Candidatus Micrarchaeota archaeon CG1 02 55 22* | 0.54863 | 0.55795 | 0.0000 | 0.0000 |
| *uncultured beta proteobacterium* | 0.54863 | 0.55795 | 0.0000 | 0.0000 |
| *Solibacillus* | 0.54863 | 0.55795 | 0.0000 | 0.0000 |
| *Sphingobacterium* | 0.54863 | 0.55795 | 0.0000 | 0.0000 |
| *Syntrophorhabdus* | 0.54863 | 0.55795 | 0.0000 | 0.0000 |
| *Sva0081 sediment group* | 0.54863 | 0.55795 | 0.0000 | 0.0000 |
| *Burkholderia-Caballeronia-Paraburkholderia* | 0.54863 | 0.55795 | 0.0000 | 0.0000 |
| *Pusillimonas* | 0.54863 | 0.55795 | 0.0000 | 0.0000 |
| *Rheinheimera* | 0.54863 | 0.55795 | 0.0000 | 0.0000 |
| *Membranicola* | 0.54863 | 0.55795 | 0.0000 | 0.0000 |
| *Achromobacter* | 0.54863 | 0.55795 | 0.0000 | 0.0000 |
| *Pajaroellobacter* | 0.54863 | 0.55795 | 0.0000 | 0.0000 |
| *Mizugakiibacter* | 0.54863 | 0.55795 | 0.0000 | 0.0000 |
| *uncultured prokaryote* | 0.54863 | 0.55795 | 0.0000 | 0.0000 |
| *Negativibacillus* | 0.54863 | 0.55795 | 0.0000 | 0.0000 |
| *Acidothermus* | 0.54863 | 0.55795 | 0.0000 | 0.0000 |
| *uncultured alpha proteobacterium* | 0.54863 | 0.55795 | 0.0000 | 0.0000 |
| *gut metagenome* | 0.54863 | 0.55795 | 0.0000 | 0.0000 |
| *uncultured Aciditerrimonas sp.* | 0.54863 | 0.55795 | 0.0000 | 0.0000 |
| *Nitrosomonas* | 0.54863 | 0.55795 | 0.0000 | 0.0000 |
| *uncultured Actinomycetales bacterium* | 0.54863 | 0.55795 | 0.0000 | 0.0000 |
| *uncultured Chloroflexi bacterium* | 0.54863 | 0.55795 | 0.0000 | 0.0000 |
| *Aeriscardovia* | 0.54863 | 0.55795 | 0.0000 | 0.0000 |
| *Lachnospiraceae ND3007 group* | 0.54863 | 0.55795 | 0.0000 | 0.0000 |
| *Chujaibacter* | 0.54863 | 0.55795 | 0.0000 | 0.0000 |
| *Alcanivorax* | 0.54863 | 0.55795 | 0.0000 | 0.0000 |
| *Chitinophaga* | 0.54863 | 0.55795 | 0.0000 | 0.0000 |
| *Comamonas* | 0.54863 | 0.55795 | 0.0000 | 0.0000 |
| *Coprobacter* | 0.54863 | 0.55795 | 0.0000 | 0.0000 |
| *SWB02* | 0.54863 | 0.55795 | 0.0000 | 0.0000 |
| *Catonella* | 0.54863 | 0.55795 | 0.0000 | 0.0000 |
| *Cryptosporangium* | 0.54863 | 0.55795 | 0.0000 | 0.0000 |
| *Candidatus Competibacter* | 0.54863 | 0.55795 | 0.0000 | 0.0000 |
| *Sinibacillus* | 0.54863 | 0.55795 | 0.0000 | 0.0000 |
| *Deinococcus* | 0.54863 | 0.55795 | 0.0000 | 0.0000 |
| *Slackia* | 0.54863 | 0.55795 | 0.0000 | 0.0000 |
| *Eggerthella* | 0.54863 | 0.55795 | 0.0000 | 0.0000 |
| *Spirosoma* | 0.54863 | 0.55795 | 0.0000 | 0.0000 |
| *Rhizocola* | 0.54863 | 0.55795 | 0.0000 | 0.0000 |
| *Bulleidia* | 0.54863 | 0.55795 | 0.0000 | 0.0000 |
| *Anaerobiospirillum* | 0.54863 | 0.55795 | 0.0000 | 0.0000 |
| *Fabibacter* | 0.54863 | 0.55795 | 0.0000 | 0.0000 |
| *Pseudonocardia* | 0.54863 | 0.55795 | 0.0000 | 0.0000 |
| *Finegoldia* | 0.54863 | 0.55795 | 0.0000 | 0.0000 |
| *Thalassobaculum* | 0.54863 | 0.55795 | 0.0000 | 0.0000 |
| *Treponema* | 0.54863 | 0.55795 | 0.0000 | 0.0000 |
| *Tumebacillus* | 0.54863 | 0.55795 | 0.0000 | 0.0000 |
| *Bdellovibrio* | 0.54863 | 0.55795 | 0.0000 | 0.0000 |
| *Turneriella* | 0.54863 | 0.55795 | 0.0000 | 0.0000 |
| *Geobacillus* | 0.54863 | 0.55795 | 0.0000 | 0.0000 |
| *Ruminococcaceae UCG-009* | 0.54863 | 0.55795 | 0.0000 | 0.0000 |
| *Arachidicoccus* | 0.54863 | 0.55795 | 0.0000 | 0.0000 |
| *Zoogloea* | 0.54863 | 0.55795 | 0.0000 | 0.0000 |
| *Anaerocella* | 0.54863 | 0.55795 | 0.0000 | 0.0000 |
| *uncultured rumen bacterium* | 0.54863 | 0.55795 | 0.0000 | 0.0000 |
| *Arthrobacter* | 0.55609 | 0.56395 | 0.0001 | 0.0002 |
| *Anaerostipes* | 0.56031 | 0.56662 | 0.0001 | 0.0000 |
| *Faecalibaculum* | 0.68757 | 0.69336 | 0.0006 | 0.0002 |
| *Lachnospira* | 0.83319 | 0.83786 | 0.0000 | 0.0000 |
| *Brevibacterium* | 0.92017 | 0.92274 | 0.0001 | 0.0001 |
| *Fusobacterium* | 0.93246 | 0.93246 | 0.0002 | 0.0002 |
